# Intestinal CD103+ Dendritic Cells Are Key Players in the Innate Immune Control of *Cryptosporidium parvum* Infection in Neonatal Mice

**DOI:** 10.1371/journal.ppat.1003801

**Published:** 2013-12-19

**Authors:** Louis Lantier, Sonia Lacroix-Lamandé, Laurent Potiron, Coralie Metton, Françoise Drouet, William Guesdon, Audrey Gnahoui-David, Yves Le Vern, Edith Deriaud, Aurore Fenis, Sylvie Rabot, Amandine Descamps, Catherine Werts, Fabrice Laurent

**Affiliations:** 1 INRA, UMR1282 Infectiologie et Santé Publique, Nouzilly, France; 2 Universite Francois Rabelais, UMR1282 Infectiologie et Sante Publique, Tours, France; 3 Institut Pasteur, Unité de Régulation Immunitaire et Vaccinologie, Paris, France; 4 Centre d'Immunologie de Marseille-Luminy, Université d'Aix-Marseille, Marseille, France; 5 INRA, UMR 1319, Micalis, Jouy-en-Josas, France; 6 Institut Pasteur, Unité de Biologie et Génétique de la Paroi Bactérienne, Paris, France; University of Geneva, Switzerland

## Abstract

*Cryptosporidium parvum* is a zoonotic protozoan parasite found worldwide, that develops only in the gastrointestinal epithelium and causes profuse diarrhea. Using a mouse model of *C. parvum* infection, we demonstrated by conditional depletion of CD11c+ cells that these cells are essential for the control of the infection both in neonates and adults. Neonates are highly susceptible to *C. parvum* but the infection is self-limited, whereas adults are resistant unless immunocompromised. We investigated the contribution of DC to the age-dependent susceptibility to infection. We found that neonates presented a marked deficit in intestinal CD103+ DC during the first weeks of life, before weaning, due to weak production of chemokines by neonatal intestinal epithelial cells (IEC). Increasing the number of intestinal CD103+ DC in neonates by administering FLT3-L significantly reduced susceptibility to the infection. During infections in neonates, the clearance of the parasite was preceded by a rapid recruitment of CD103+ DC mediated by CXCR3-binding chemokines produced by IEC in response to IFNγ. In addition to this key role in CD103+ DC recruitment, IFNγ is known to inhibit intracellular parasite development. We demonstrated that during neonatal infection CD103+ DC produce IL-12 and IFNγ in the lamina propria and the draining lymph nodes. Thus, CD103+DC are key players in the innate immune control of *C. parvum* infection in the intestinal epithelium. The relative paucity of CD103+ DC in the neonatal intestine contributes to the high susceptibility to intestinal infection.

**Authors Summary**

Dendritic cells are central to the defense against mucosal pathogens. They are numerous and form a uniform network in the intestinal mucosa of adults, but are poorly characterized in the intestine of neonates. Young animals are more susceptible than adults to intestinal pathogens, such as *Cryptosporidium parvum*, a zoonotic agent distributed worldwide that develops in the epithelium of the small intestine causing profuse diarrhea. We show that dendritic cells are scarce in the small intestine of neonates until weaning and that increasing their numbers *in vivo* results in increased resistance to infection. Using a conditional depletion model we demonstrate that the presence of dendritic cells is necessary for the control of the infection in both neonates and adults. During infection in neonates, dendritic cells are rapidly recruited into the intestine by chemokines produced by the epithelium and produce interferon gamma, a cytokine that inhibits parasite development in epithelial cells. Thus, the low number of dendritic cells in the intestinal mucosa of neonates is responsible for their sensitivity to cryptosporidiosis, and probably contributes to the general susceptibility of neonates to intestinal diseases.

## Introduction

*Cryptosporidium parvum* is a waterborne protozoan parasite. It is highly prevalent worldwide affecting primarily populations in underdeveloped countries but also causes disease in industrialized countries such as the US where there are approximately 748,000 cryptosporidiosis cases annually [1]. Infection of the intestinal epithelium by this zoonotic agent results in sickness and severe diarrhea that can be life threatening in very young children and ruminants. Immunocompetent adults are relatively resistant to the infection but immunosuppressed individuals, particularly those with HIV infection, are particularly susceptible [2]. As for humans and ruminants, age-related differences in susceptibility are observed in the mouse model of infection used to study the immune mechanism leading to protection. The severity of this infection is related to the immune status of its host. Unlike other intestinal parasites, such as *Toxoplasma gondii*, *C. parvum* is only minimally invasive and its development throughout its life cycle is restricted to the epithelial layer. Therefore, in addition to its economic and clinical importance, it can serve as a model for studies of the immune mechanisms protecting the neonatal epithelium.

Neonates are generally more susceptible than adults to infectious diseases [3]. Their intestinal immune system is in development and subject to numerous changes after birth, facing the colonization by the commensal flora, alimentary antigens, and aggression by enteric pathogens [3]. Both qualitative and quantitative differences between the neonatal and adult immune systems have been documented [4]. Several factors in the intestine can contribute to neonatal susceptibility to infections; they include the thinner than adult mucous layer, low level of epithelial proliferation, low alpha defensin production, and lower level of expression or specific compartmentalization of various TLRs [5]. In addition, the numbers of resident lamina propria and intraepithelial T lymphocytes are low at birth although they increase thereafter [6]. Neonatal mononuclear phagocytes have been characterized in human cord blood and in the spleen of mice [7], but much less is known about the presence of the subsets associated with the intestinal mucosa in neonates. After a long debate, the situation concerning the nature and the origin of the different intestinal CD11c+ cell subsets in adult mice has been clarified based on the expression of the fractalkine receptor, CX3CR1, and the integrin alpha-E (CD103) [8]. Conventional dendritic cells (cDC) originate from pre-cDC precursor from the blood and express CD103+, whereas CD11c+CX3CR1+ is a heterogeneous population, originating from Ly6c+ precursors, and most are now referred to as macrophages [9].

In addition to their well-described role in antigen presentation and lymphocyte activation, DC play an active role in innate immunity against infection by protozoans via their crosstalk with other innate cells such as NK cells [10] and as a critical source of cytokines [11]. We previously observed a rapid control of *C. parvum* development in neonates receiving TLR-receptor ligand treatment, evidence of the importance of innate immune responses to the control of this infection [12]. We and others have observed that following administration of immunostimulants or during the natural course of *C. parvum* infection, IFNγ is an essential component in the mechanism of protection [12]–[16] but the nature of the cells producing this cytokine in the intestine of neonatal mice has yet to be identified.

We investigated the reasons for the neonatal susceptibility to *C. parvum* infection and the immune mechanism leading to protection against the acute phase of this infection. We show that CD103+ DC are scarce in the small intestine of neonates, and this results in high susceptibility to *C. parvum* infection. Accordingly, *in vivo* amplification of CD103+ DC in neonates resulted in increased resistance to the infection. These cells are actively recruited during the natural infection by CXCR3-binding chemokines and independently of conventional T cells and conventional NK cells (cNK), participate in the control of parasite development. Thus, CD103+ DC act as gatekeepers to control infections of the neonatal intestinal epithelium by enteric pathogens.

## Results

### Intestinal CD11c+ cells are necessary to control *C. parvum* infection whatever the age of the animal

**Figure 1:**
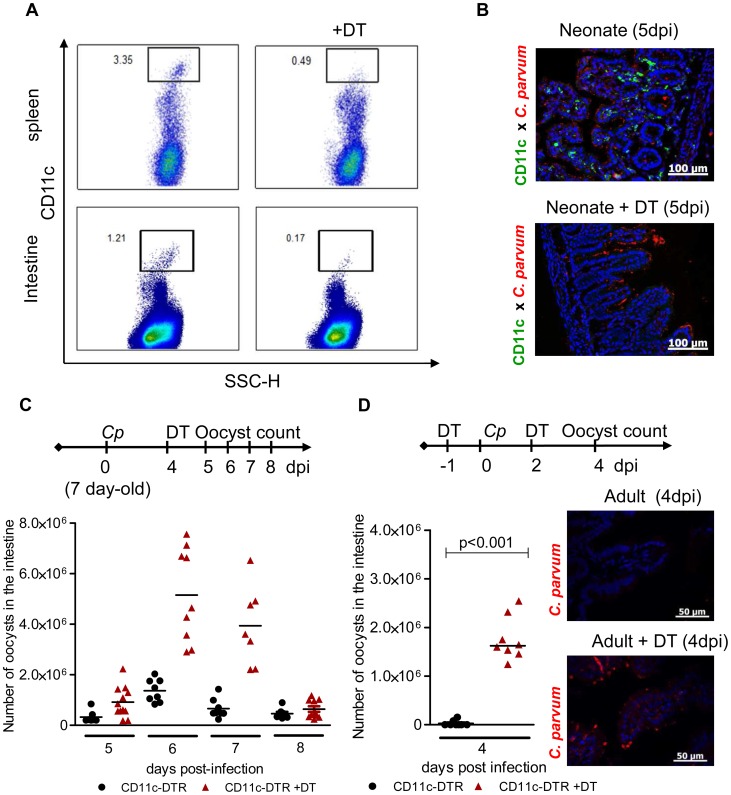
CD11c+ cells are necessary for the control of *C. parvum* infection. (**A**) Seven day-old heterozygous CD11c-DTR neonates were infected with 5.10^5^ *C. parvum* oocysts and some animals were treated with DT 4 dpi. Spleen cells and intestinal cells were collected separately 24 hours later and CD11c+ cell depletion was analyzed by flow cytometry. (**B**) Neonates were infected and treated as in (A), and sections of the small intestine from CD11c-DTR neonates collected 5 dpi were processed for histology: *C. parvum* is stained in red, and CD11c is stained in green (Original magnification ×200). (**C**) Neonates were infected and treated as in (A). The parasite load was assessed daily from 5 dpi to 8 dpi in CD11c-DTR neonates treated or not treated with DT 4 dpi (n = 7–12 neonatal mice per group). (**D**) Adult CD11c-DTR were infected with 10^6^ *C. parvum* oocysts and treated twice (d−1; d+2) or not treated with DT. Parasite load was evaluated 4 dpi in the whole intestine of adult mice (p<0.001, n = 8 mice per group). In the same experiment, histological sections of small intestine were collected 4 dpi (*C. parvum* staining in red; original magnification ×200).

We previously observed a rapid control of *C. parvum* development in neonates receiving strong immunostimulant treatment, evidence of the importance of innate immune responses to the control of this infection [12]. In addition to their role in the orchestration of adaptive immune responses, DC participate in innate responses to pathogens by producing cytokines [17]. To study the contribution of intestinal CD11c+ cells to protection against *C. parvum*, we used the CD11c-DTR mouse model that allows transient depletion of CD11c+ cells after diphtheria toxin (DT) administration [18]. The DT dose (2 ng/g) was adapted to neonates and was sufficient to deplete CD11c+ cells efficiently in both systemic (>85%) (Figure 1, A) and intestinal (>86%) compartments (Figure 1, A and B). When DT was injected once 4 days post inoculation, a clear increase in parasite load was observed reaching a peak 48 h later (Figure 1, C). New CD11c+ cells emerged and colonized the mucosa such that their numbers were similar to those in non DT treated animals 72 h post-treatment (Figure S1, A), and the infection started to decrease rapidly thereafter. Adult C57BL/6 mice are naturally resistant to *C. parvum* infection, with oocysts barely detectable in the feces. Adult CD11c-DTR mice were depleted of CD11c+ cells by intraperitoneal injections with 4 ng/g of DT 12 h preceding the infection and 2 dpi. In the intestine 4 dpi, the depletion of CD11c+ cells was at least 85% (Figure S1, B) and this was associated with numerous parasites found in the intestinal content and throughout the ileal epithelium (Figure 1, D). Overall, these experiments indicate a strong correlation between the presence of CD11c+ in the intestinal mucosa and the ability to control *C. parvum* infection. In addition to CD103+ DC that were efficiently depleted by DT treatment in infected neonatal mice (5 dpi, >91%), other mononuclear phagocytes express CD11c+ in the intestine [9]; however, the relative contribution of these CD11c+ cell populations to the mechanism of protection at this stage could not be assessed.

### The neonatal mouse intestine is almost devoid of CD11c+ CD103+ DC but these cells are recruited strongly during the course of infection

Quantitative differences in immune cell composition in peripheral tissues between neonates and adults have been reported [4], [6]. To test for the influence of age-dependent colonization by mononuclear phagocytes on the susceptibility to infection, we compared the presence in the small intestinal mucosa of various subsets of mononuclear phagocytes in 7-day-old and 13-day-old neonates and in adult mice. In the adult intestine, it is possible to distinguish CX3CR1^−^CD103^+^ DCs and an heterogeneous population of CX3CR1^int^CD103^−^ mononuclear phagocytes, which have characteristics intermediate between those of DCs and macrophages, and a population of bona fide CX3CR1^hi^ F4/80^+^ macrophages [9], [19].

**Figure 2:**
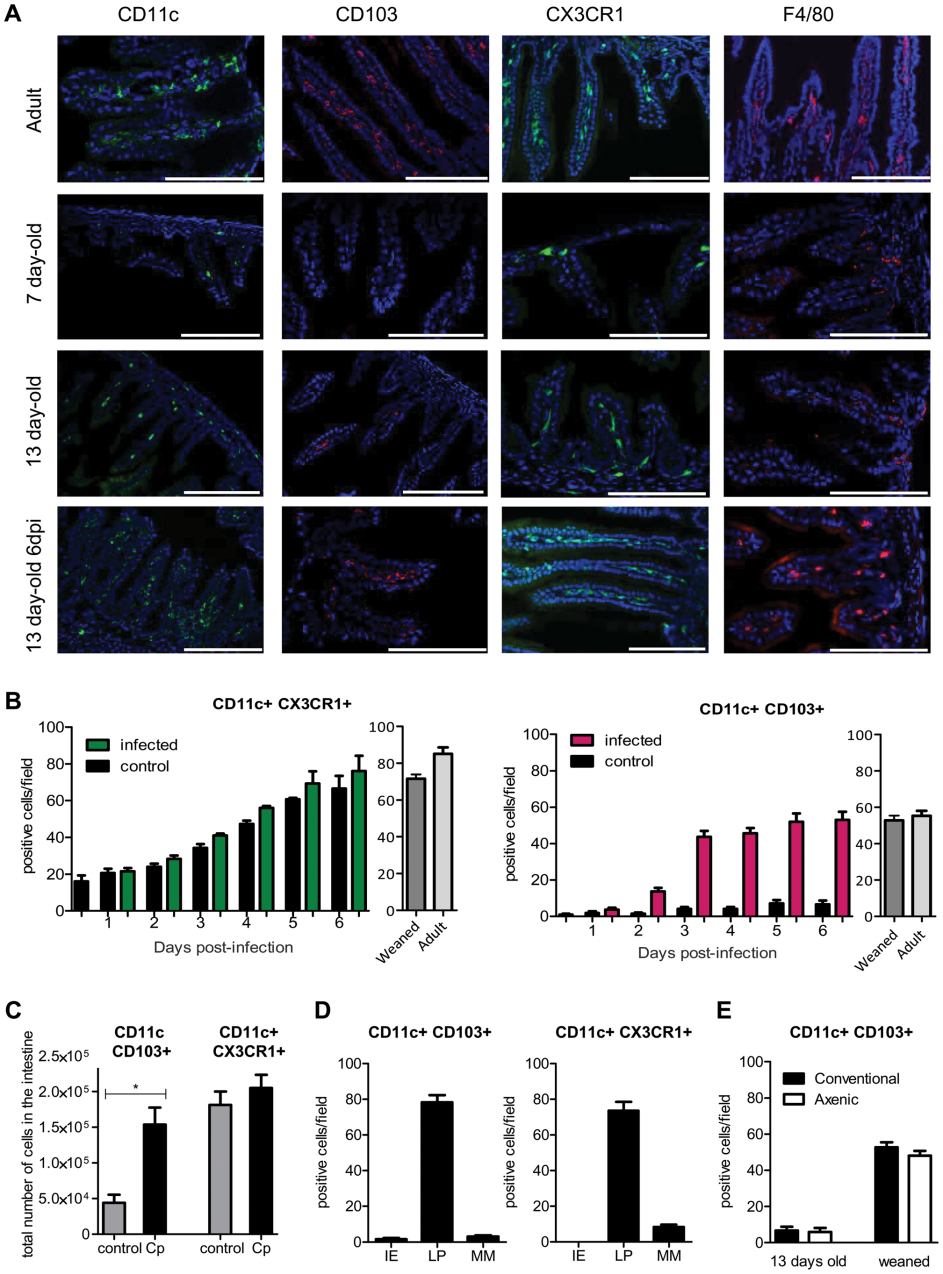
Rapid recruitment of CD11c+ CD103+ in the infected mucosa. (**A**) Sections of the small intestine of mice at different ages: 7 days old, 13 days old, 13 days old inoculated with *C. parvum* at 7 days of age, and uninfected adult mice were stained with Hoechst dye and antibodies against CD11c, CD103 and F4/80. CX3CR1^GFP/WT^ mice were used for CX3CR1 detection. Original magnification ×200, scale bars indicate 100 µm. Double staining for CD11c+ CD103+, CD11c+ CX3CR1+ and F4/80+ CX3CR1+ are provided in Figure S2. (**B**) Number of CD11c+CX3CR1+ and CD11c+ CD103+ double-positive cells in the intestine of infected animals and controls. (**C**) CD11c+ CX3CR1+ and CD11c+ CD103+ double-positive cells were quantified by flow cytometry for infected animals 6 dpi and for age matched controls (n = 5 animals per group). Gating strategies are provided in Figure S5, A. (**D**) As in (B) but the location in the mucosa of CD11c+ CX3CR1+ and CD11c+ CD103+ double-positive cells was evaluated in the intestine of infected animals 6 dpi. IE: intraepithelial; LP: Lamina propria; MM: Muscularis mucosae. (**E**) As in (B), CD11c+ CD103+ double-positive cells were enumerated in the intestine of axenic or conventional neonates at age 13 days and post weaning (22 days old). For (B, D, E) the values reported were obtained by counting double-positive cells in sections of the small intestine of mice and for each point are the means ± SEM of at least 30 optical fields from two animals from different litters.

The two main non overlapping subsets, CD11c+CD103+ and CD11c+CX3CR1+, formed a well-developed network within the lamina propria of the small intestine of adult mice. In one week-old healthy neonates, mononuclear phagocytes were much less abundant in the small intestine (Figure 2, A). At that age, CD11c+ cells were almost undetectable, and F4/80+ macrophages were present in small numbers and mainly within the *intestinal muscularis*. The few CX3CR1+ cells that were present in the lamina propria of neonates (Figure 2, A) also expressed F4/80+ and were the first resident macrophages (data not shown). Thirteen-day-old neonates were still almost devoid of intestinal CD11c+ CD103+ DC under homeostatic conditions (8 times less than in adults). Daily analysis during the course of infection revealed that CD11c+ CX3CR1+ macrophage-like cells continuously accumulated such that the count increased 3 fold between the first and second week of age, to reach a density close to that in 22-day-old and adult animals (Fig 2, B). The infection had only a limited effect on the numbers of CD11c+ CX3CR1+ cells per villus. Strikingly, the situation was completely different for CD11c+CD103+ DC: they were rare in the intestinal mucosa until weaning (day 22) but were observed in very large numbers after *C. parvum* infection (Figure 2, B and Figure S2, A). These results were confirmed by flow cytometry 6 dpi, at the peak of the infection (Figure 2C). The location within the lamina propria of CD11c+CD103+ DC and CD11c+ CX3CR1+ cells at 6 dpi was investigated by immunohistochemistry (Figure 2D). After infection, only very few CD11c+ cells were detected in intraepithelial positions and the few that were present were CD103+. These cells may correspond to the CD103+ DC that have been recently shown to patrol among enterocytes of the small intestine and which are able to extend dendrites toward the lumen [20]. The vast majority of CD11c+ CD103+ and CD11c+ CX3CR1+ were in the lamina propria close to the epithelium.

The neonatal period is associated with massive colonization by the intestinal flora, and this increases the number of CD11c+ CX3CR1+ cells in the adult intestine [21]. We investigated the role of the intestinal flora in the recruitment of CD103+ DC between 13 and 22 days of age. Germ-free neonatal mice presented similar numbers of CD11c+CD103+ DC in their intestine as conventional animals (Figure 2, E), indicating that this recruitment was independent of the intestinal flora. Between 2 and 3 dpi, CD103+DC counts increased rapidly in the small intestine. GM-CSF and especially FLT3-L are important hematopoietic factors for the differentiation of CD103+ DC [22]. We analyzed their expression in the infected mucosa 2 dpi but found no significant difference with age-matched controls (Figure S2, B). Therefore, this result suggests that rather than a local expansion, the large number of CD103+ DC in the infected intestine was due to the migration of blood precursors (pre-DC) into the intestinal tissue, as previously reported for CD103+ DC in adults [23].

### *In vivo* amplification of CD103+ DC by repeated FLT3-L administration increases resistance to the infection

We tested whether the low frequency of CD11c+ in the neonatal intestinal mucosa was responsible for the susceptibility to *C. parvum* infection. Bone marrow DC differentiated with GMCSF or with FLT3-L were transferred into day-old neonates through the superficial temporal vein. Despite a successful transfer to the circulating blood, these cells were not found in the intestine and the parasite load after challenge was unaffected (Figure S3). Due to the small size of the animals, the small numbers of DC that could be transferred (2×10^5^) may have been limiting.

**Figure 3:**
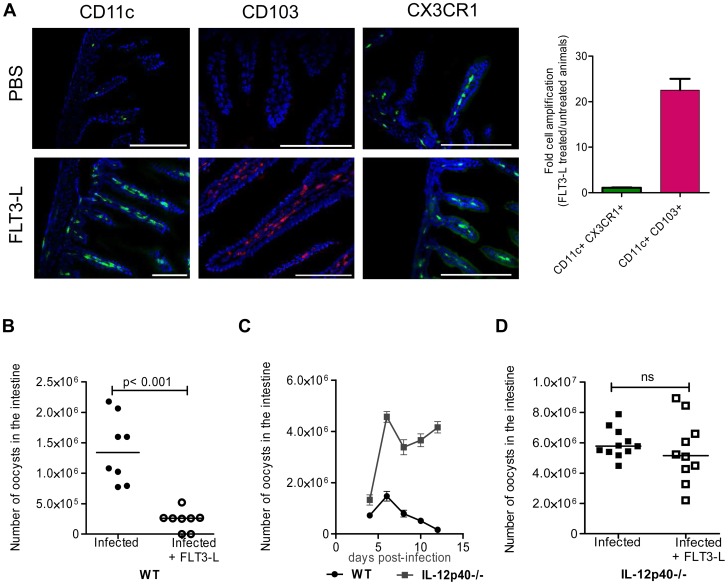
*In vivo* amplification of CD11c+ CD103+ DC by FLT3-L enhances neonatal resistance to *C. parvum* infection. (**A**) WT and CX3CR1^GFP/WT^ neonates were injected daily for 6 consecutive days from birth with 1 µg FLT3-L. The first three injections were subcutaneous and the next three intraperitoneal. Histological sections of the ilea were collected from FLT3-L-treated or untreated mice at 7 days of age. Sections were stained with Hoechst dye and antibodies against CD11c and CD103. CX3CR1^GFP/WT^ mice were used for CX3CR1 detection. Single staining is shown, to provide a clearer overview of the distribution of the positive cells in the villi (scale bars indicate 100 µm). Double positive CD11c+CX3CR1+ cells and CD11c+ CD103+ DC in ileal sections were counted. The graph reports means ± SEM of at least 20 optical fields from two different animals giving similar results; the values are fold amplification (treated/untreated animals). (**B**) Control neonates and neonates treated with FLT3-L for 6 days were orally infected with 5.10^5^ oocysts of *C. parvum* at 7 days of age and the parasite load was evaluated 6 dpi. Values are means ± SEM (p<0.001, n = 8 neonatal mice per group). (**C**) Seven day-old WT and IL-12p40−/− neonates were orally infected with 5.10^5^ oocysts of *C. parvum* and the parasite load was evaluated at various times post infection. Values are means ± SEM (n = 5–8 neonatal mice per group at each time point). (**D**) Same experiment as in (B) but with IL12p40−/− neonates. Values are means ± SEM (ns: not significant, n = 10–11 neonatal mice per group). The Mann-Whitney non-parametric analyses were considered significant when p values were less than <0.05. Figure 3D, p>0.05 is non-significant (ns).

We therefore increased the number of CD103+ DC present by administering several injections of FLT3-L from birth to age 6 days, according to a previously published protocol [24]. On day 7, the frequency of CD11c+CD103+ DC within the lamina propria was very much higher than that in controls. This population was more numerous than that of CD11c+CX3CR1+ cells, the expression of which is independent of FLT3-L [25] and therefore not affected by the treatment (Figure 3, A). FLT3-L treatment did not significantly increase the number of NKp46+NK1.1+ cNK and CD3+ lymphocytes (data not shown). FLT3-L-treated neonates were infected with *C. parvum*, and the parasite development 6 dpi was severely reduced (80% lower than in controls; Figure 3, B). These data confirm that the presence of CD103+ DC in the mucosa of neonates is essential for controlling the initial infection and its subsequent development. FLT3-L increases IL-12 secretion by stimulated DC [24]; we therefore investigated the contribution of IL-12 to the mechanism of protection. We observed that IL-12p40 is necessary to control the acute phase of the infection in neonates (Figure 3, C), as previously described in adults [25]. The IL-12p40−/− neonatal mice that received FLT3-L showed higher CD103+ DC counts (21±4 fold amplification), similar to those in wild-type mice, in the lamina propria of the small intestine. However, despite this large number of CD103+DC, these neonates were unable to control the infection in the absence of IL-12p40 (Figure 3, D). The kinetics of *C. parvum* infection is similar in IL12-p35−/− mice that cannot produce IL-12 and IL-12p40−/− mice that cannot produce both IL-12 and IL-23 [26]. Moreover, in our experimental conditions, we never detected IL-23p19 chain upregulation during the infection (data not shown). Thus, functional IL-12 is required for the control of *C. parvum* infection.

### Chemokine mRNAs are less abundant in the intestinal epithelial cells of neonates than adults

**Figure 4:**
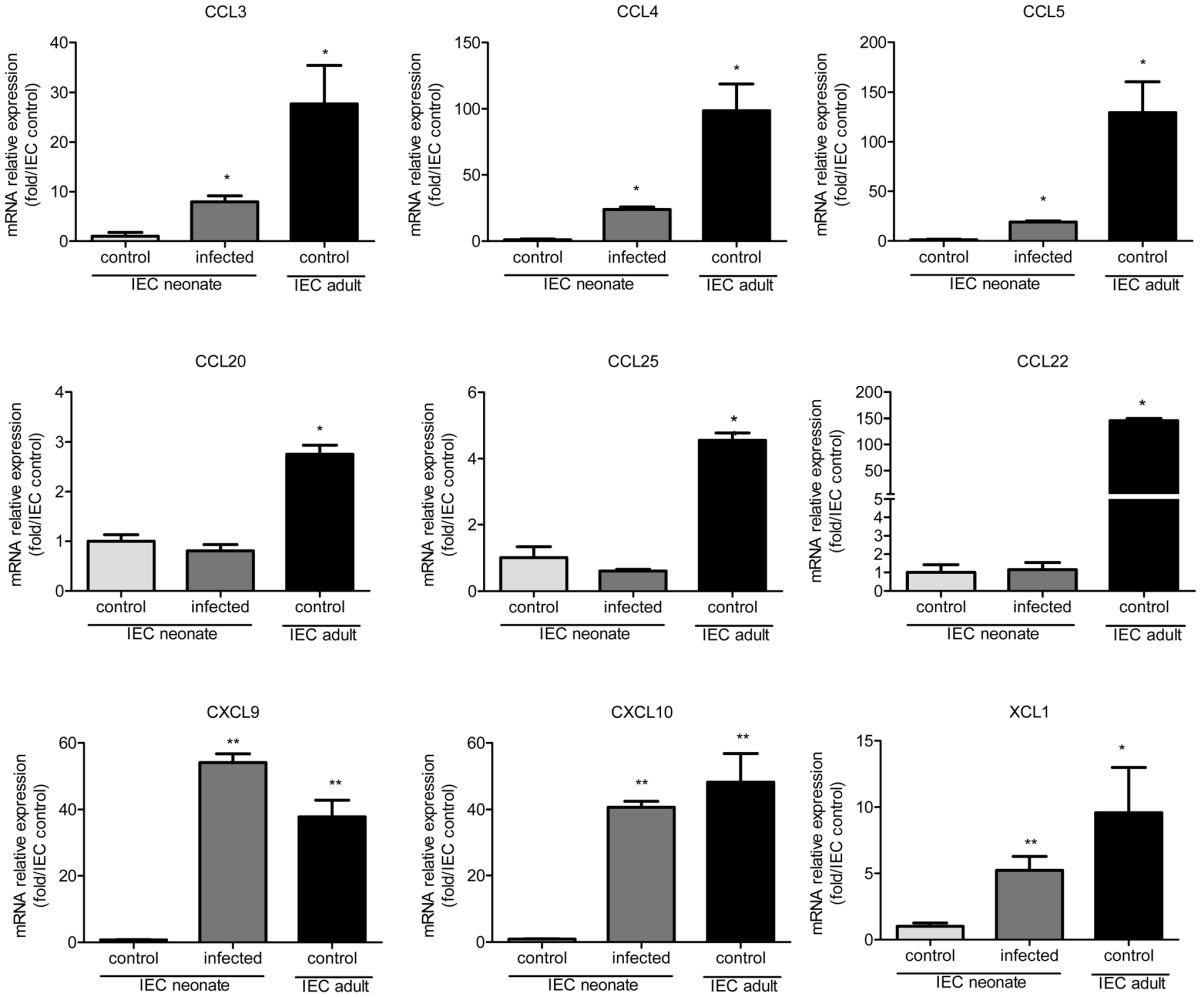
Weak chemokine expression by epithelial cells of neonates is upregulated by the infection. RNA was extracted from intestinal epithelial cells isolated from 13 day-old control neonates, infected (13 day-old, 6 dpi) neonates and control adult mice. Chemokine mRNAs were assayed by qRT-PCR. Values were all normalized to IEC isolated from control neonates (*p<0.05, **p<0.01; n = 4–6 in each group).

IEC produce chemokines that attract DC [27], [28]. We investigated if differences in chemokine production between neonatal and adult epithelium contribute to the differential intestinal colonization by CD103+ DC. We found that in IEC from healthy animals, the mRNAs for DC-attracting chemokines were less abundant in 13 day-old neonates than adults. This was particularly marked for CCL3, CCL4, CCL5, CCL22, CXCL9 and CXCL10 for which the differences were one or two logs (Figure 4). IEC isolated from neonates at the peak of the infection presented a clear up-regulation of XCL1, CCL3, CCL4, CCL5, CXCL9, and CXCL10 expression, with levels close to those in adults for CXCL9 and CXCL10.

### CXCR3 controls by an IFNγ-dependent mechanism the strong recruitment of CD103+ dendritic cells in the intestine of neonates infected by *C. parvum*

**Figure 5:**
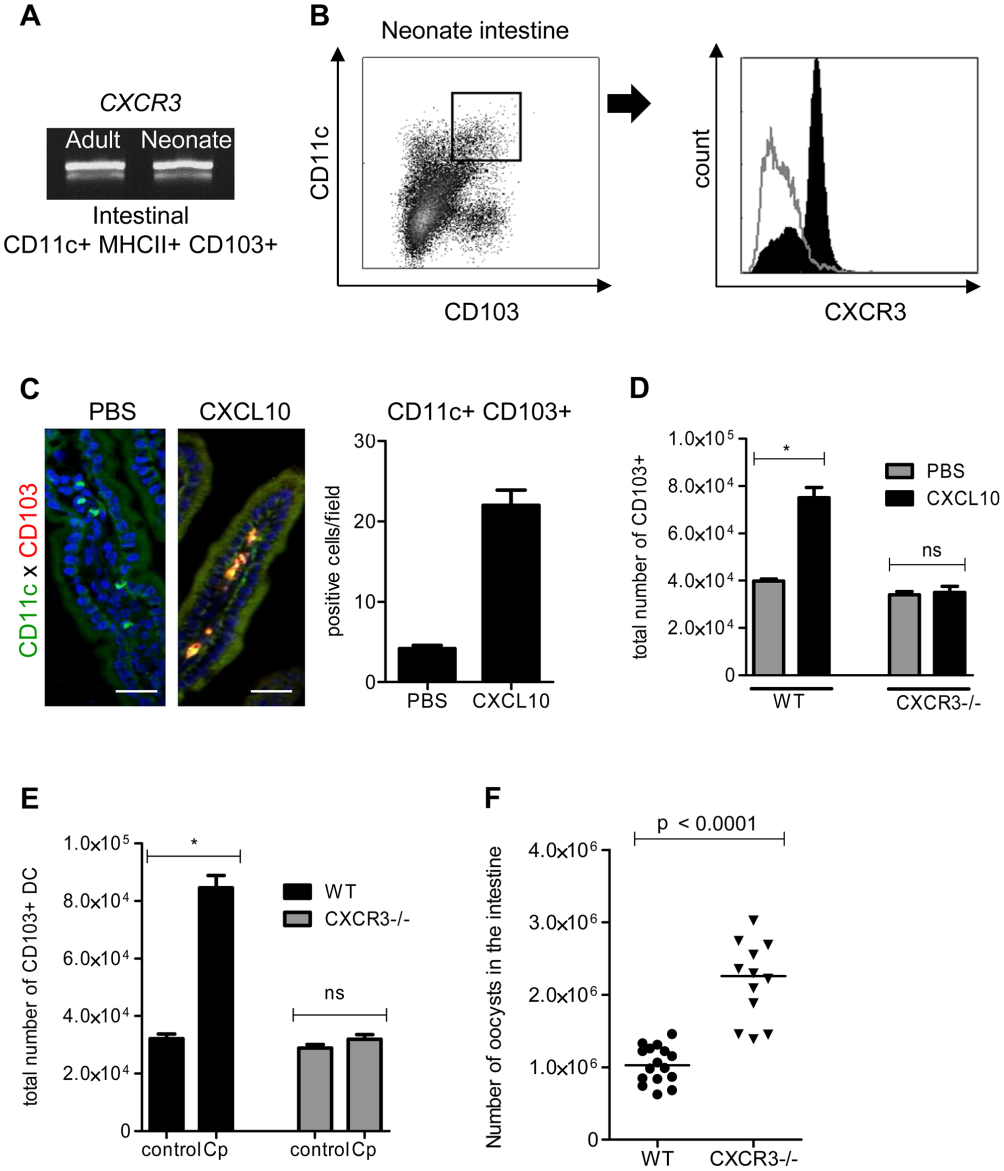
The chemokine receptor CXCR3 plays a major role in the recruitment of CD103+ DC during *C. parvum* infection. (**A**) CD11c+ MHCII+ CD103+ DC isolated from the intestines of uninfected adults and infected neonates were sorted by flow cytometry. CXCR3 expression in each sample was evaluated by RT-PCR (amplicons on a 2% agarose gel). (**B**) Surface expression of CXCR3 on intestinal CD11c+CD103+ DC (previously gated on MHCII+ cells) isolated from infected neonates (6 dpi) was analyzed by flow cytometry. Gating strategies are provided in Figure S5, B. The grey line histogram represents the isotype control and the black filled histogram the staining with anti-CXCR3 monoclonal antibody. (**C**) Recombinant CXCL10 was administered (1 µg/per os) to 7 day-old neonates for 3 consecutive days starting from day 7. At 9 days of age, CD11c+ CD103+ labeling and counting were performed on intestinal sections. CD11c+ cells are stained in green, CD103+ cells in red and nuclei in blue with Hoechst dye (scale bars indicate 20 µm). Data in the right-hand panel were obtained by counting double-positive cells per field in sections of the small intestine. Reported values are means ± SEM of at least 30 optical fields from two neonates from different litters. (**D**) CD11c+ MHCII+ CD103+ DC recruitment after CXCL10 treatment in WT and CXCR3−/− neonates was also analyzed by flow cytometry. Numbers of CD11c+MHCII+CD103+ cells in the small intestine in treated and untreated neonates (*p<0.05, n = 4 neonates for each group) are shown. (**E**) Seven day-old WT and CXCR3−/− neonates (*p<0.05, n = 4 neonates for each group) were infected with *C. parvum* and CD103+ DC recruitment was analyzed by flow cytometry 3 dpi. Gating strategies are provided in Figure S5, B. (**F**) Seven day-old WT and CXCR3−/− neonates were infected with *C. parvum* and the parasite load was evaluated 6 dpi. (p<0.0001, n = 12–16 mice per group).

To identify the mechanism of recruitment of CD103+ DC in the infected mucosa, we performed functional studies *in vivo* with CXCL10: CXCL10 was one of the most strongly upregulated chemokines in the IEC during neonatal mouse infection and the mRNA for its receptor, CXCR3, was found to be expressed in CD103+ DC isolated from neonates and adult mice (Figure 5, A). Expression of CXCR3 on cell surfaces was confirmed by flow cytometry with CD11c+MHCII+CD103+ DC isolated from the intestine of neonatal mice at the peak of the infection (Figure 5, B). The oral administration of CXCL10 for 3 consecutive days starting from day 7 to neonates, that express this chemokine poorly in their IEC, induced the recruitment of numerous CD103+ DC in the mucosa (Figure 5, C). The specificity of the mechanism of recruitment was confirmed with CXCR3−/− neonatal mice: oral administration of CXCL10 to these mice did not induce CD103+ DC recruitment in the intestine (Figure 5, D). To further validate CXCL10 as a candidate for the recruitment of CD103+ DC, we analyzed their recruitment in the intestine of CXCR3−/− neonates at the early stage of the infection (3 dpi). CD103+ DC were not significantly recruited in the intestine of these neonates compared to their WT counterparts (Figure 5, E). Finally to evaluate the importance of CXCR3 in the mechanism of protection CXCR3−/− neonatal mice were infected with *C. parvum*. These neonatal mice presented a significantly higher level of infection than controls at the peak (6 dpi) of the infection (Figure 5, F). Overall these data demonstrate that CXCR3 is an important receptor for neonatal CD103+ DC colonization during *C. parvum* infection.

**Figure 6:**
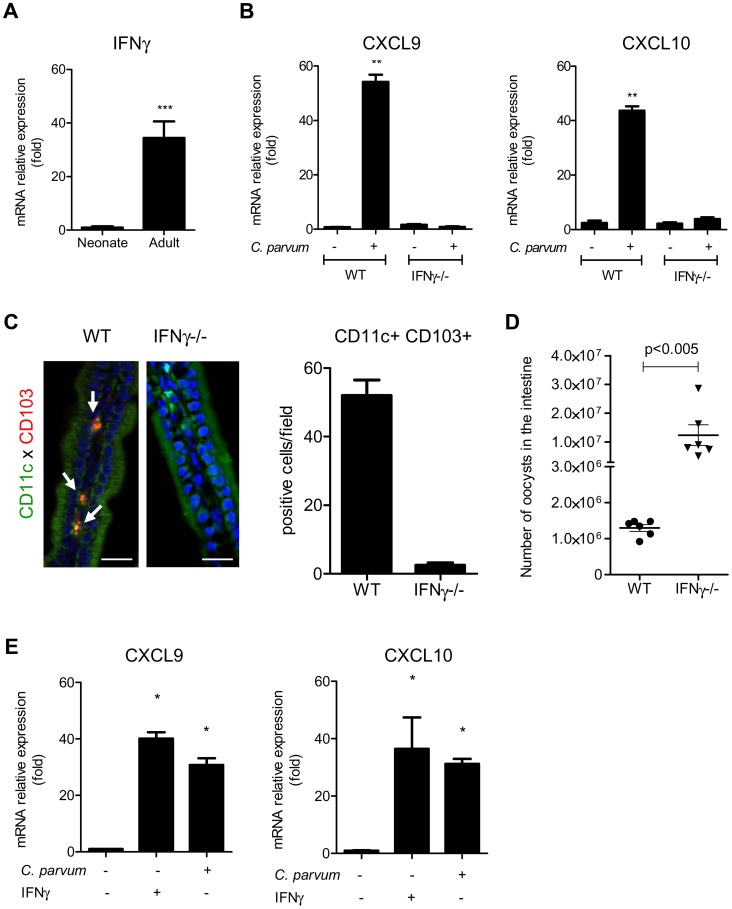
IFNγ plays a key role in the recruitment of CD103+DC during infection. (**A**) Basal mRNA level of IFNγ in the intestine of neonate and adult mice (p<0.001, n = 8 mice per group). (**B**) Seven day-old C57BL/6J WT and IFNγ−/− neonates were infected with *C. parvum*. mRNAs for CXCL9 and CXCL10 in isolated IEC were assayed by qRT-PCR in infected (6 dpi) and in uninfected age-matched control neonates (n = 6 neonatal mice for each group,** p<0.005). (**C**) The presence of CD11c+CD103+ DC was evaluated in the ileal villi of WT and IFNγ−/− mice 6 dpi. Sections of small intestine were immunostained and CD11c+ CD103+ double-positive cells (white arrow) were counted. CD11c+ cells are stained in green, CD103+ cells in red and nuclei in blue with Hoechst dye (scale bars indicate 20 µm). Data in the right-hand panel were obtained by counting double-positive cells per field in sections of the small intestine of C57BL/6J WT and IFNγ−/− mice at 6 dpi. Reported values are means ± SEM of at least 30 optical fields from two neonates from different litters. (**D**) Seven day-old WT and IFNγ−/− neonates were infected with *C. parvum*. The parasite loads 6 dpi in WT and IFNγ−/− neonatal mice are reported. Values represent means ± SEM (n = 6 neonatal mice per group). (**E**) CMT-93 cells were left unstimulated or stimulated with IFNγ (10 ng/ml) or infected with *C. parvum* at a ratio of three oocysts/cell. Expression of CXCL9 and CXCL10 mRNAs was analyzed by qRT-PCR 24 h later (*p<0.01 relative to control CMT-93 cells).

CXCL9 and CXCL10 bind to CXCR3 and their expression is dependent on IFNγ. Under steady state conditions, there was around 30 times less mRNA for IFNγ in the intestinal mucosa of neonates than adults (Figure 6, A) explaining the weaker production of CXCL9 and CXCL10. Accordingly, IEC isolated from IFNγ−/− neonatal mice did not significantly upregulate the expression of CXCL9 and CXCL10 during the infection (Figure 6, B) whereas other chemokines such as CCL3, CCL4 and CCL5 were still upregulated (Figure S4, A). In IFNγ−/− neonatal mice, the recruitment of CD103+DC was dramatically altered (Figure 6, C) despite a higher level of infection (Figure 6, D). Overall these findings show that the initial low number of CD103+DC was linked to the lower basal expression of IFNγ in the neonatal intestine and consequently to low CXCL10-related chemokine production. Following infection by *C. parvum* sporozoites *in vitro*, the mouse epithelial cell line CMT-93 upregulated CXCL9 and CXCL10 24 h later (Figure 6, E) suggesting that independently of IFNγ, IEC can in some conditions upregulate these chemokines in direct response to the parasite. However, our findings with IFNγ−/− neonatal mice show that during the infection CXCL9 and CXCL10 upregulation was strongly IFNγ-dependent. IFNγ therefore plays a key role in the mechanism of recruitment of CD103+ DC in the infected mucosa.

### Neonatal CD103+ dendritic cells produce IL-12 and IFNγ in response to the infection

**Figure 7:**
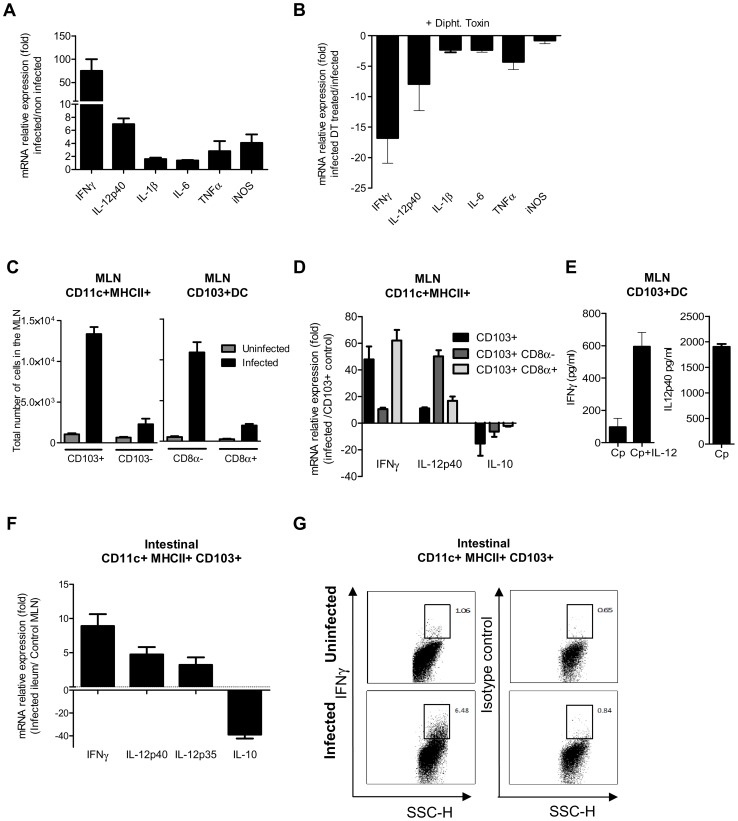
CD103+ dendritic cell subsets contribute to IL-12p40 and IFNγ production. (**A**) Seven day-old neonates were infected with *C. parvum* and ilea were collected 6 dpi for mRNA extraction and subsequent qRT-PCR. For each gene, mRNA expression is represented as a fold increase with respect to uninfected neonates. The values are means ± SEM (n = 6 neonatal mice per group). (**B**) Gene expression after CD11c+ cell depletion was analyzed by injecting DT into CD11c-DTR neonates at 4 dpi. RNA was extracted from the ileum 6 dpi. For each gene, mRNA abundance is represented as fold decrease relative to untreated neonates. The values reported are means ± SEM (n = 6 neonatal mice per group). (**C**) Total numbers of CD11c+MHCII+CD103+ in the MLN of infected (6 dpi) and age matched control neonates (13-day-old) in the left panel. Data from the same experiment, in the right panel, show the total numbers of CD8α+ and CD8α− CD103+ DC subsets (n = 4 pools, 2–3 neonates/pool). (**D**) isolation of CD103+ cell subsets from the MLN of neonates was first based on selection of CD11c^hi^ MHC II^hi^. Double-positive cells were further separated into two subsets based on CD8α expression. Gating strategies are provided in Figure S5, C. CD103+ cells, CD103+ CD8α− and CD103+ CD8α+ subsets were isolated from pooled MLN obtained 6 dpi from numerous neonates (2–4 pools, 6–44 neonates per pool). CD103+ DC were also isolated from their age-matched controls (2 pools, 72 neonates per pool) for normalization. Isolated cells were used for qRT-PCR analysis. Gene expression in pools of samples was assessed by qRT-PCR. For each gene, mRNA expression in the different subsets isolated from infected animals is represented as fold differences to that in CD103+ DC from control neonates. The reported values are means ± SEM. (**E**) MLN were isolated from infected neonates 6 dpi, and CD11c+ MHCII+ CD103+ DC were sorted by FACS and cultured *in vitro*. After 24 h of culture, supernatants were assayed by ELISA for IFNγ and IL-12p40 (n = 5 pools, 2 neonates/pool). Some cells were cultured in the presence of 10 ng/ml of IL-12 and IFNγ production assayed. (**F**) CD103+ CD11c+ MHC II+ DC were isolated from the intestine of infected neonates. Due to the low frequency of CD11c+CD103+ DC in the intestine of uninfected neonates at 13 days of age, CD11c+CD103+ DC were isolated from their MLN for normalization. RNA extraction and qRT-PCR were performed immediately after cell sorting. Gating strategies are provided in Figure S5, B. The reported values are means ± SEM (6 pools of neonatal mice per group; between 8–21 neonates per pool). (**G**) Intracellular staining of IFNγ in samples from infected (6 dpi) and uninfected animals (13 day-old). Panels show intracellular staining of IFNγ in CD11c+MHCII+CD103+ DC (panels are representative of two independent experiments).

To identify the mechanism by which CD103+ DC control parasite multiplication, we investigated the expression of cytokines in the mucosa of WT neonates in the presence, or soon after depletion, of CD11c+ cells. DC can produce NO [29] and previous finding with neonatal iNOS−/−mice suggested that NO may help to control *C. parvum* infection [30]. iNOS expression was therefore also analyzed. The expression of cytokines and iNOS in the mucosa of WT neonates was studied 6 dpi. There was modest upregulation of the mRNAs, with the exception of IFNγ that was upregulated by up to 100 fold and to a lesser extent IL-12p40 that was upregulated 7 fold (Figure 7, A). When CD11c+ cells were depleted, the upregulation of IL-12p40 and IFNγ resulting from the infection was severely impaired (Figure 7, B).

We next investigated the cytokines expressed by the two subsets of CD103+ DC that were recently described in the intestine on the basis of CD8α expression [31]. After mucosal activation, CD11c+ CD103+ DC from the intestinal tissues migrate to the mesenteric lymph node (MLN) via the lymphatic system to serve classical dendritic cell functions [8]. We observed that among CD11c+MHCII+ cells at the peak of the infection, CD103+ DC numbers had increased substantially in the MLN, with CD103+CD8α− DC being the predominant subset over the CD103+CD8α+ DC subset (Figure 7C). We first analyzed the cytokine expression by CD103+ CD8α+ and CD103+ CD8α− DC isolated from the MLN at 6 dpi. Analysis of the mRNAs showed clear upregulation of IFNγ and IL-12p40 in both CD103+ subsets of DC isolated from infected animals (Figure 7, D). The CD103+ CD8α− subset presented the greatest IL-12p40 upregulation, and expression of IFNγ by CD103+ CD8α+ DC was increased by about 60 fold. Moreover, a decrease in IL-10 mRNA levels was observed in both subsets. The production of both IFNγ and IL-12p40 proteins by CD103+ DC of MLN from infected animals was confirmed by ELISA (Figure 7 E). This IFNγ production was further amplified (6 fold) by the addition of exogenous IL-12 to the culture media (Figure 7 E).

The small representation of CD103+ DC in the intestine of 13 day-old uninfected neonates precluded the isolation of these cells. However, by using numerous infected neonates, we succeeded in isolating a sufficient number of CD103+ DC for qRT-PCR analysis. CD103+ DC isolated from the MLN of uninfected neonates were used as a reference and therefore the gene expression evaluations are only indicative. CD103+ DC isolated from the intestine of infected neonates expressed IFNγ, IL-12p40 and IL-12p35, but much a lower level of IL-10 than CD103+DC isolated from the MLN of uninfected neonates (Figure 7, F). CD11c+MHCII+CD103+ cells isolated from the small intestine of infected (6 dpi) and uninfected age-matched controls were stained for IFNγ and this confirmed that a fraction of CD103+DC do indeed produce IFNγ during infection (Figure 7, G).

### Neither conventional Natural Killer cells nor conventional T lymphocytes are major players in the control of the acute phase of the infection

**Figure 8:**
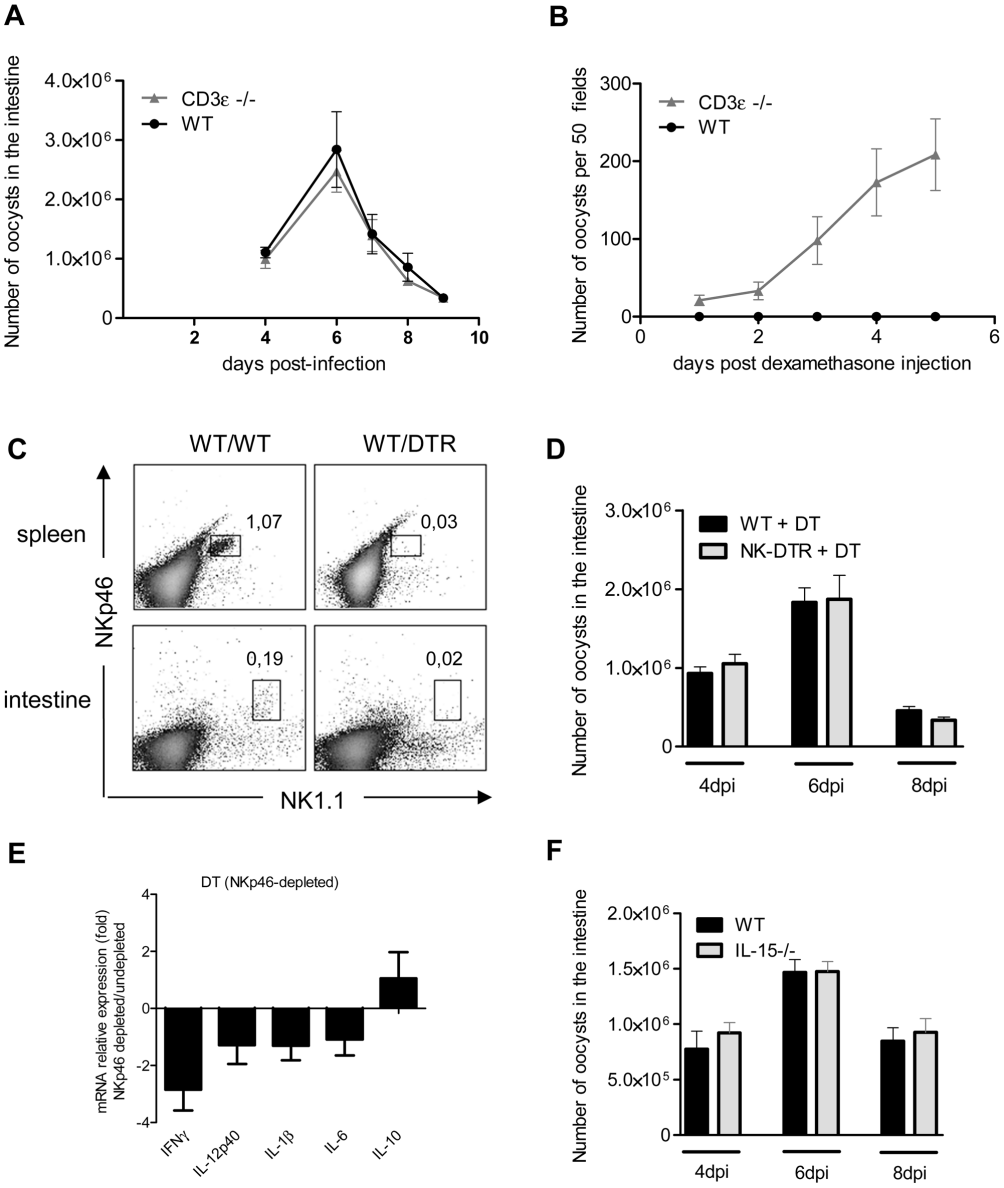
NKp46+NK1.1+ Natural killer cells and conventional T cells are not essential for the control of the acute phase of *C. parvum* infection. Seven day-old neonates were orally infected with 5.10^5^ *C. parvum* oocysts and the parasite load in the whole intestine was evaluated at various times post infection. (**A**) Parasite load was evaluated in CD3ε−/− and WT neonates. Data are means ± SEM (n = 6 neonatal mice per group). (**B**) Four weeks after infection of CD3ε−/− and WT neonates, and apparent recovery from the infection, mice were injected with 1 µg of dexamethasone, by the IP route, daily for 3 days. Fecal smears were used to quantify oocyst excretion every day. Data are means ± SEM (n = 5 mice per group). (**C**) Flow cytometry analysis of NKp46+NK1.1+ cells in the intestine and spleen of infected heterozygous NKp46-DTR neonates and infected wild-type littermates, all treated with DT on d−1; d+1 and d+3 post infection. The results shown are for representative animals from the same litter. (**D**) Same experiment as in (C). The parasite load was evaluated at 4, 6 and 8 dpi. Data are means ± SEM (n = 6–15 neonatal mice per group). (**E**) Similar experiment as described in (C) and cytokine mRNAs in the ilea of neonates 6 dpi were analyzed by qRT-PCR. The bars represent the mean values ± SEM of the ratios of the relative expression value for mRNA in the intestine of NKp46-depleted neonatal mice to that in non-depleted wild type littermates (n = 6). (**F**) Parasite load was evaluated 4, 6 and 8 dpi in IL15−/− and WT neonates. Data are means ± SEM (n = 6 neonatal mice per group).

Dendritic cells cross-talk with both innate and T lymphocytes to control many intracellular pathogens. We first studied the role of conventional T lymphocytes in the control of *C. parvum* infection in CD3ε-deficient mice (CD3ε−/−). The parasite loads during the acute phase of the infection in CD3ε−/− neonates were similar to those in wild-type neonates (Figure 8, A). However, following dexamethasone-induced immunosuppression 4 weeks after infection, only CD3ε−/− mice excreted *C. parvum* oocysts revealing that the infection was not definitively cured in the absence of functional conventional T cells (Figure 8, B).

Previous studies have suggested a role for NK cells in innate immunity to *C. parvum* [32], [33]. Conventional NK cells (cNK) express the natural cytotoxicity triggering receptor NKp46 and NK1.1 in C57BL/6 mice. We first tested for the presence of NKp46+NK1.1+ cells in our cell preparation isolated from the intestine of neonates at 6 dpi. Only a small double positive NKp46+NK1.1+ population could be observed, whereas these cells were clearly identified in the spleen of the same animals (Figure 8, C). In NKp46-DTR animals [34] NKp46+NK1.1+ cells were depleted by DT-treatment but these neonatal mice presented no difference in susceptibility to infection (Figure 8, D). In the same conditions, the level of IFNγ expression observed in the intestine of DT treated NKp46-DTR neonatal mice was only (2.7 fold) modestly decreased (Figure 8, E). IL-15−/− mice lack conventional NK cells (cNK). In agreement with our NKp46 depletion data, IL-15−/− neonatal mice exhibited parasite loads similar to those in controls at the various time points tested (Figure 8, F). Overall these data suggest that cNK cells expressing both NK1.1 and NKp46 are not a major contributor in the mechanism of protection.

## Discussion

*Cryptosporidium* infection is most prevalent among children below 5 years of age and in neonatal ruminants. The disease is of substantial medical and economic importance. As the infection is restricted to the intestinal epithelium and is strongly dependent on the immune status of the host it is a useful model for deciphering the immune mechanism protecting the intestinal epithelium. Previously, only fragmented information was available regarding the immune mechanisms leading to protection against this zoonotic parasite.

Different patterns of expression of receptors involved in parasite penetration may contribute to the difference between neonatal and adult sensitivity to the parasite. However, *C. parvum* develops successfully in immunodeficient adults suggesting that susceptibility depends on immune responses. Neonatal sensitivity to infection has been attributed to both qualitative and quantitative differences in immune cell components [4]. The numbers of intraepithelial and lamina propria lymphocytes in the intestine of mice [6] and calves [35] are low at birth and increase progressively over the first three weeks. In our mouse model of infection, we demonstrated that conventional CD3+ lymphocytes are not essential for the control of the acute phase of the infection, but are important for subsequent sterilizing immunity. Our results are in agreement with Korbel *et al.* who did not observe any exacerbation of *C. parvum* infection in neonatal WT mice depleted of CD4(+) T cells [36]. Other quantitative differences may explain neonatal sensitivity to cryptosporidiosis. We have indeed observed a pronounced defect of all mononuclear cells in the neonatal intestine. Although the density of various subsets of macrophages expressing F4/80 and/or CX3CR1 increases steadily in the intestinal mucosa during the first weeks after birth, the profound deficit of CD103+ DC was maintained until weaning. A recent study in which chlodronate liposomes were administered to chronically infected adult mice suggested that phagocytic cells may be involved in the mechanism of protection, but the nature of the depleted cells was not investigated [37]. We showed that following CD11c+ cell depletion, neonates and adults become highly susceptible to the infection, revealing the crucial role of these cells in controlling parasite replication in enterocytes. Amplification of the number of intestinal CD103+ DC resident *in vivo* by administration of FLT3-L was associated with increased resistance to the infection. Therefore, discovering the mechanism governing the recruitment of these cells may allow the development of strategies to strengthen intestinal immune defenses.

In uninfected animals, we found that the lower than adult basal level of IFNγ in the neonatal mucosa is associated with weaker expression of CXCL9 and CXCL10, and this explains the poor colonization by CD103+ cells that express CXCR3. In the absence of functional CXCR3, CD103+DC recruitment during neonatal *C. parvum* infection is severely impaired. Zeng *et al.* recently showed that most pre-μDC that express gut homing receptors and gave rise to intestinal cDC also express CXCR3 [38]. Indeed, the oral administration of CXCL10 to neonatal mice that possess functional CXCR3 leads to rapid recruitment of CD103+DC in the mucosa; this reveals that there is no major defect in either the capacity of neonatal pre-DC to respond to chemokine gradients or their binding to vascular addressins on the endothelium and subsequent extravasation to gut mucosal tissues.

Following infection *in vitro*, *C. parvum* sporozoites induce the production of CXCL9 and CXCL10 in IEC by a yet-unidentified mechanism. NF-kB binding sites are present in the CXCL10 gene promoter [39] so this chemokine production may be related to the NF-kB activation observed after infection [40], [41]. Previous work in our laboratory showed that CCR5−/− neonatal mice presented a higher parasite burden at the early stage of infection but eliminated the parasite as efficiently as their wild-type counterparts [42]. CCL3, CCL4 and CCL5 are produced by IEC independently of IFNγ and CCR5 is expressed by CD103+ DC (Figure S4, B), so these chemokines may provide the first signals for CD103+ DC recruitment during *C. parvum* infection. The substantial reduction of CD103+ DC recruitment in infected IFNγ−/− neonatal mice demonstrates that CXCL9 and CXCL10 are responsible for the high level of recruitment of CD103+DC. With CXCR3−/− neonatal mice, we demonstrated the importance of chemokine/CXCR3 interactions for controlling parasite replication. In humans, high levels of CXCL10 are produced by IEC in AIDS patients with active cryptosporidiosis [43]; following effective antiparasite and antiretroviral therapy, *Cryptosporidium* infections resolve, and the levels of CXCL10 decrease to normal. In addition, CXCR3 is expressed on CD141^hi^ DC that are functionally homologous to mouse CD103+ non lymphoid DC [44]. These are important observations that suggest that a similar mechanism of DC recruitment occurs in humans during cryptosporidiosis. Our experiments involving FLT3-L injection into IL-12p40−/− mice demonstrated that the presence of numerous CD103+ DC in the mucosa is not sufficient to control *C. parvum* infection; control was critically dependent on the host capacity to produce IL-12. In IL-12p40−/− neonatal mice, the strong expression of IFNγ observed in WT neonates was dramatically impaired (data not shown). Therefore, CD103+DC —the major IL-12 producers in the infected mucosa— play a key role in IFNγ production. Adult mice in which IFNγ expression or its signalling are affected such as IFNγ−/− or STAT-1−/− mice [26] display high sensitivity to the infection and long-term carriage of the parasite. The presence of the IFNγ receptor at the basolateral surface of the enterocyte makes this cytokine a key final mediator of the immune mechanism of protection. IFNγ is identified as being the most effective cytokine for controlling parasite replication in enterocytes despite the immuno-evasive strategy employed by the parasite based on depletion of the STAT1a protein [45]. Despite intensive investigations, the cells producing IFNγ in the intestinal mucosa during neonatal cryptosporidiosis have not been identified.

NK cells through direct cell-to-cell contact and IL-12 production by DC can be major producers of IFNγ and therefore may be effector cells. Previous studies suggest that NK cells may participate in the control of *C. parvum* infection. An *in vitro* study showed that IL-15-activated human NK cells upregulate NKG2D and lyse infected HCT-8 target epithelial cells expressing the MHC I-related molecules MICA and MICB [33]. However, neither IL-15−/− neonatal mice nor neonatal mice depleted of NKp46+ cells displayed enhanced susceptibility. In mice, NK1.1 is present on the surface of subsets of NKT and innate lymphoid cells, such as cNK cells [46], [47]. Korbel *et al.* showed that neonatal mice treated with anti-NK1.1 antibody at the time of the infection and at 3 dpi had significantly larger numbers of *C. parvum* oocysts, as determined by counts obtained from fecal smears [36]. One possible explanation for the discrepancy between these results is that NKp46 and NK1.1 are expressed by both common and different populations of innate lymphocytes. The depletion of NKp46 or NK1.1 may therefore result in different outcomes of *C. parvum* infection.

The dramatic downregulation of IFNγ after CD11c+ depletion but not NKp46+ depletion suggested that DC may themselves produce this cytokine. Moreover, these cells are numerous in the infected mucosa and can be found in close contact with epithelial cells making DC a candidate for controlling infected enterocytes. We discovered that CD103+ DC isolated from the intestine and MLN of infected neonates produce IFNγ. Therefore, by producing IL-12, CD103+ DC subsets may mediate their own IFNγ production via both paracrine and autocrine mechanisms. IFNγ-producing DC have been identified in few other adult mouse models of infection or gastrointestinal inflammation. Moretto *et al.* reported that IFN-gamma-producing DC are important for priming the gut intraepithelial lymphocyte response against intracellular parasitic infection with *Encephalitozoon cuniculi* [48]. Also, CD103+ DC were recently identified as a significant source of IFNγ in the MLN of mice fed a vitamin A-deficient diet [49]. In addition, Sun *et al.* showed that CD11c+ DC from neonatal spleen produce more IFNγ than their adult counterparts, in particular when stimulated with IL-12 [50]. This suggests that the capacity of neonatal mouse DC to produce IFNγ may be greater than that of their adult counterparts.

However, our data do not exclude the possibility that there is another source of IFNγ such as unconventional T cells and/or innate lymphoid cells that could be stimulated by the IL-12 produced by CD103+ DC. To assess this possibility, further investigations would be required, including characterization of these cell types in the intestinal mucosa of neonates and their relative significance in the control of *C. parvum* infection.

Our work highlights the major role of the cytokine response of CD103+DC in controlling the acute phase of the infection. It would be now very informative to investigate how *C. parvum* antigen is captured and presented to T cells in the MLN to trigger the adaptive sterilizing immunity. In addition to antigen transfer from intestinal CX3CR1+ cells to CD103+DC [51], [52], a recent 2-photon microscopy study demonstrates that CD103+DC can efficiently phagocytize bacteria using intraepithelial dendrites [20]. This mechanism may also be relevant to enteric protozoans such as *C. parvum*.

Overall, the investigations we report substantially improve the characterization of the mechanisms involved in protection against *C. parvum*, in particular by identifying the critical role of intestinal CD103+ DC. The poor colonization of the neonatal intestine by CD103+ DC explains the neonatal sensitivity to *C. parvum* infection, a phenomenon that probably also contributes to susceptibility to other intestinal pathogens. In addition, their role in the induction of adaptive responses, CD103+ DC can thus be considered to be a sentinel population that contributes to the protection of the epithelium via innate mechanisms. By describing the precise mechanism by which these CD103+DC can be recruited in the neonatal intestine, we also provide a basis for further development of immunomodulatory DC-based strategies to protect neonates against enteric infections.

## Materials and Methods

### Ethics statement

All experimental protocols were conducted in compliance with French legislation (Décret: 2001-464 29/05/01) and EEC regulations (86/609/CEE) governing the care and use of laboratory animals, after validation by the local ethics committee for animal experimentation (CEEA VdL): 2011-12-6; 2011-04-06; 2011-09-11.

### Mice

IFNγ^−/−^, IL12p40^−/−^, CD3ε^−/−^, IL15^−/−^,CXCR3−/−, CX3CR1^GFP^, CD11c-DTR and NK-DTR mice, all in a C57BL/6 background, were maintained in the animal facilities of the PFIE (INRA-Tours) in accordance with European guidelines. Mice were maintained under specific pathogen-free conditions at constant temperature and humidity, with food and water given *ad libitum*. NK-DTR and CD3ε−/− mice were provided by Eric Vivier (CIML, France) and Armelle Phalipon (Institut Pasteur, France), respectively. CXCR3−/− mice were provided by Christophe Combadière (UPMC, France). CD11c-DTR (EM 00044) and CX3CR1-GFP (EM 00055) mice were provided by the European Mouse Mutant Archive (EMMA).

### Parasite and mouse infection

*C. parvum* oocysts were initially isolated from the feces of an infected child and were maintained by repeated passage in neonatal calves. Oocysts were purified as previously described [53]. Neonatal mice were infected with 5×10^5^ oocysts by the oral route at 7 days of age. Adult CD11c-DTR mice were infected with 10^6^ oocysts by the oral route. The level of infection in individual animals was assessed by counting oocysts in the intestinal content. Whole intestines were individually homogenized in 1 ml of water with an Ultra-Turrax. Oocysts were then counted in Sheather's solution using a Thoma cell chamber. After dexamethasone treatment of adult mice, oocyst numbers in Ziehl-Neelsen stained fecal smears were counted under the microscope.

### Experimental protocols with mice

For CD11c and NKp46 depletion, CD11c-DTR and NKp46-DTR transgenic neonatal mice were injected intraperitoneally with 2 ng/g and 4 ng/g, respectively, body weight of DT (Servibio) on the days indicated in the figure legends. Adult CD11c-DTR mice were injected with 4 ng/g body weight of DT.

### Reagents

Recombinant FLT3-L and recombinant CXCL10 were from eBioscience. For histological studies, cells were labeled with the following antibodies: anti-CD11c (HL3), anti-CD103 (M290), anti-F4/80 (CI:A3-1), anti-hamster-IgG alexa488 and anti-rat alexa594 (Invitrogen). For FACS analyses, the following antibodies were used at 1 µg/10^6^ cells: anti-CD11c APC (N418), anti CD11cPE (N418), anti-CD103 PE (2E7), anti-CD8α APC-H7 (53-6.7), anti-IA/IE FITC (2G9), anti CXCR3 FITC (CXCR3-173), anti NKp46 APC (9E2), anti NK1.1 PE(PK136), anti IFNγPECy7 (4S.B3), anti-CD16/CD32 (2.4G2). For *C. parvum* staining, we generated a rat polyclonal antiserum against oocyst antigens.

### Immunofluorescent staining

Immunofluorescence histology was performed as previously published [54]. Eight µm-thick sections were stained with antibodies and secondary antibodies in 1% BSA, 0.1% Triton. Slides were Hoechst stained and mounted in Fluoromount medium (Interchim). Separate images were collected at 200× magnification for each fluorochrome and overlaid to obtain multicolor images with Axiovision software (Zeiss) and final processing of the images was performed with Photoshop software (Adobe).

### Cell preparation, flow cytometry and cell sorting, and ELISA

MLN cells and IEC from adults or neonates were prepared as previously described [53]. For intestinal DC isolation, entire intestines were cut longitudinally and into 0.5 cm pieces, pooled and washed with 1% penicillin/streptomycin in PBS. Tissues were then incubated three times in HBSS without calcium and magnesium, with 5% FCS and 5 mM EDTA at 37°C for 15 minutes with gentle shaking to remove epithelial cells. Pieces of intestine were then incubated with shaking in HBSS with calcium and magnesium containing 5% FCS, collagenase (100 U/ml) and DNase-I (100 µg/ml) for 1 h at 37°C. Supernatants were passed through 60 µm-pore size filters and the collected cells washed with 10% FCS in PBS. These isolated cells were first stained with anti-CD16/CD32 antibody in FACS medium (PBS, 1% FCS, 2 mM EDTA) and with the various antibodies. Cells were analyzed on a *FACSCalibur* flow cytometer (Beckton Dickinson) with the CellQuest-Pro software and further analyzed with FSC Express3 software. CD11c+ subsets were sorted with a High Speed Fluorescence Activated Cell Sorter (Beckman Coulter) and cell subsets were immediately used for RNA extraction. For intracellular staining of IFNγ, intestinal cells were incubated with Brefeldin A for 4 hours (3 µg/ml) immediately after isolation, and before surface and intracellular staining. The Fixation/Permeabilization Kit (BD Biosciences) was used prior to intracellular staining of IFNγ. ELISA (mouse IFN-gamma or IL-12p40 DuoSet (R&D)) were performed with aliquots of 10^5^ sorted cells cultured for 24 h in p96 wells. For IFNγ production, IL-12 (10 ng/ml) was added to some wells.

### RNA extraction and reverse transcription (RT)-PCR analysis

RNA extraction from the ileum of neonates and reverse transcription were performed as previously described [12]. RNA from isolated DC was extracted with PicoPure kits (ARCTURUS). Real-time RT-PCRs were run on a Bio-Rad Chromo4 (Bio-Rad). Results were normalized to the three most suitable reference genes (HPRT, TBP, PPia) selected from five using geNorm [55]. Gene expression values are expressed as relative values after Genex macro analysis (Bio-Rad).

### Statistics

The Mann-Whitney test was used for non-parametric analyses. P values of less than <0.05 were considered significant.

## Supporting Information

Figure S1 **Restoration of CD11c+ cell counts in the infected mucosa of CD11c-DTR neonatal mice after transient depletion with DT.** The presence of CD11c+ cells in the intestinal tissue was analyzed by flow cytometry. (**A**) Seven day-old heterozygous CD11c-DTR neonates were infected with 5.10^5^ *C. parvum* oocysts and some animals were treated with DT 4 dpi. Intestinal cells were purified at 48 h, 72 h and 96 h post DT-treatment. (B) Adult CD11c-DTR animals were treated with DT and intestinal CD11c+ cells analyzed 24 h later.
(TIF)

Figure S2 **Mononuclear phagocyte populations in the intestinal mucosa of neonates at the peak of** ***C. parvum*** **infection.** (**A**) Sections of the small intestine of infected neonates (6 dpi) were stained with Hoechst stain and antibodies against CD11c, CD103 and F4/80. CX3CR1GFP/+mice were used for CX3CR1 detection. The white arrows in the merge panel indicate double-positive cells. Intestinal CD103+ cells are distinct from F4/80+ cells such as CD11c+ cells and F4/80+ cells (Original magnification ×200; scale bars indicate 20 µm). (**B**) M-CSF, FLT3-L and GMCSF mRNAs were assayed in the ilea of 9 day-old neonates infected or not infected at 7 days of age. Data are means ± SEM of at least eight neonates in each group. Differences were not significant (ns) as assessed by Mann-Whitney non-parametric analyses (p values>0.05).
(TIF)

Figure S3 **Dendritic cell transfer to neonates.** (**A**) BMDC and FLDC were generated *in vitro* with GM-CSF and FLT3-L, respectively. Cells were injected by the intravenous route through the superficial temporal vein (see white arrow) according to Sands and Barker (Sands and Barker, 1999). Aliquots of 2×10^4^ stained BMDC were injected into day-old neonates; at that age, the skin is transparent, and the needle is visible through the skin. (**B**) For *in vivo* tracking of transferred cells, BMDC were stained with PKH67 and the presence of positive cells in the total spleen cell population of recipient neonates was analyzed 24 h (same results at 48 h) after the IV injection. The boxed region in the graph represents CD11c+ DC stained with PKH67 that have been transferred to recipient neonates. The image on the right-hand side shows a PKH67-stained cell adjacent to two unstained cells from a recipient neonate 24 h after transfer (scale bar indicates 10 µm). (**C**) Day-old littermate neonates were inoculated iv with 2×10^5^ BMDC or FLDC or mock inoculated. At seven days of age, the animals were all infected with 5×10^5^ oocysts of *C. parvum* and the parasite load in the intestine was evaluated 6 dpi. There was no significant difference between the groups. (**D**) To verify that BMDC efficiently migrated to the intestine, we tested for PKH67-BMDC in the intestine at the peak of infection by performing fluorescent microscopic analyses on sections. Despite extensive searching, no PKH67-BMDC were found in the infected intestine. Hoechst staining of the nucleus in blue (scale bar indicates 50 µm). Sands, M.S., and Barker, J.E. (1999). Percutaneous intravenous injection in neonatal mice. Lab Anim Sci 49, 328–330.
(TIF)

Figure S4 **Expression of the chemokines CCL3, CCL4, CCL5 in IEC of infected IFNγ−/− neonatal mice, and expression of CCR5 by intestinal CD1O3+ DC.** (**A**) Seven day-old C57BL/6J WT and IFNγ−/− neonates were infected with *C. parvum*. The mRNAs for CCL3, CCL4 and CCL5 in isolated IEC were assayed by qRT-PCR in infected (6 dpi) and in uninfected age-matched control neonates (n = 6 neonatal mice for each group,*** p<0.001, ** p<0.01, *p<0.05). (**B**) CD11c+ MHCII+ CD103+ DC isolated from the intestines of uninfected adults and infected neonates were sorted by flow cytometry. CCR5 expression in each sample was evaluated by RT-PCR.
(TIF)

Figure S5 **Gating strategies.** (**A**) Gating strategies for cytometry analysis of intestinal CD11c+ CD103+ cells and CD11c+CX3CR1+ of Figure 2 (**B**) gating strategies used for flow cytometry analysis of intestinal CD103+ DC provided in figure 5D, 5E, 7F and 7G. CD103+ DC analysis was first based on selection of CD11c^hi^ MHC II^hi^ cells, which were then separated according to CD103 expression as indicated. (**C**) Gating strategies used for analysis of MLN CD103+DC subsets (relative to Figure 7C, 7D). Cells were first selected based on CD11c^hi^ MHC II^hi^ gating, then separated according to CD103 and CD8α expression as indicated.
(TIF)

## References

[1] Scallan E, Hoekstra RM, Angulo FJ, Tauxe RV, Widdowson MA, et al (2011) Foodborne illness acquired in the United States−major pathogens. Emerg Infect Dis 17: 7–15. 21192848 10.3201/eid1701.P11101 PMC3375761

[2] Chen XM, Keithly JS, Paya CV, LaRusso NF (2002) Cryptosporidiosis. N Engl J Med 346: 1723–1731. 12037153 10.1056/NEJMra013170

[3] Stockinger S, Hornef MW, Chassin C (2011) Establishment of intestinal homeostasis during the neonatal period. Cell Mol Life Sci 68: 3699–3712. 21952827 10.1007/s00018-011-0831-2 PMC11114965

[4] Adkins B, Leclerc C, Marshall-Clarke S (2004) Neonatal adaptive immunity comes of age. Nat Rev Immunol 4: 553–564. 15229474 10.1038/nri1394

[5] Renz H, Brandtzaeg P, Hornef M (2012) The impact of perinatal immune development on mucosal homeostasis and chronic inflammation. Nat Rev Immunol 12: 9–23. 10.1038/nri3112 22158411

[6] Steege JC, Buurman WA, Forget PP (1997) The neonatal development of intraepithelial and lamina propria lymphocytes in the murine small intestine. Dev Immunol 5: 121–128. 9587712 10.1155/1997/34891 PMC2275980

[7] Willems F, Vollstedt S, Suter M (2009) Phenotype and function of neonatal DC. Eur J Immunol 39: 26–35. 19137537 10.1002/eji.200838391

[8] Schulz O, Jaensson E, Persson EK, Liu X, Worbs T, et al (2009) Intestinal CD103+, but not CX3CR1+, antigen sampling cells migrate in lymph and serve classical dendritic cell functions. J Exp Med 206: 3101–3114. 20008524 10.1084/jem.20091925 PMC2806467

[9] Bain CC, Scott CL, Uronen-Hansson H, Gudjonsson S, Jansson O, et al (2013) Resident and pro-inflammatory macrophages in the colon represent alternative context-dependent fates of the same Ly6C(hi) monocyte precursors. Mucosal Immunol 6: 498–510. 22990622 10.1038/mi.2012.89 PMC3629381

[10] Newman KC, Korbel DS, Hafalla JC, Riley EM (2006) Cross-talk with myeloid accessory cells regulates human natural killer cell interferon-gamma responses to malaria. PLoS Pathog 2: e118. 17154717 10.1371/journal.ppat.0020118 PMC1687207

[11] Mashayekhi M, Sandau MM, Dunay IR, Frickel EM, Khan A, et al (2011) CD8alpha(+) dendritic cells are the critical source of interleukin-12 that controls acute infection by Toxoplasma gondii tachyzoites. Immunity 35: 249–259. 21867928 10.1016/j.immuni.2011.08.008 PMC3171793

[12] Barrier M, Lacroix-Lamande S, Mancassola R, Auray G, Bernardet N, et al (2006) Oral and intraperitoneal administration of phosphorothioate oligodeoxynucleotides leads to control of Cryptosporidium parvum infection in neonatal mice. J Infect Dis 193: 1400–1407. 16619188 10.1086/503748

[13] Lean IS, McDonald SA, Bajaj-Elliott M, Pollok RC, Farthing MJ, et al (2003) Interleukin-4 and transforming growth factor beta have opposing regulatory effects on gamma interferon-mediated inhibition of Cryptosporidium parvum reproduction. Infect Immun 71: 4580–4585. 12874337 10.1128/IAI.71.8.4580-4585.2003 PMC165998

[14] Theodos CM, Sullivan KL, Griffiths JK, Tzipori S (1997) Profiles of healing and nonhealing Cryptosporidium parvum infection in C57BL/6 mice with functional B and T lymphocytes: the extent of gamma interferon modulation determines the outcome of infection. Infect Immun 65: 4761–4769. 9353062 10.1128/iai.65.11.4761-4769.1997 PMC175683

[15] Lacroix S, Mancassola R, Naciri M, Laurent F (2001) Cryptosporidium parvum-specific mucosal immune response in C57BL/6 neonatal and gamma interferon-deficient mice: role of tumor necrosis factor alpha in protection. Infect Immun 69: 1635–1642. 11179338 10.1128/IAI.69.3.1635-1642.2001 PMC98067

[16] Pollok RC, Farthing MJ, Bajaj-Elliott M, Sanderson IR, McDonald V (2001) Interferon gamma induces enterocyte resistance against infection by the intracellular pathogen Cryptosporidium parvum. Gastroenterology 120: 99–107. 11208718 10.1053/gast.2001.20907

[17] Foti M, Granucci F, Ricciardi-Castagnoli P (2004) A central role for tissue-resident dendritic cells in innate responses. Trends Immunol 25: 650–654. 15530834 10.1016/j.it.2004.10.007

[18] Jung S, Unutmaz D, Wong P, Sano G, De los Santos K, et al (2002) In vivo depletion of CD11c+ dendritic cells abrogates priming of CD8+ T cells by exogenous cell-associated antigens. Immunity 17: 211–220. 12196292 10.1016/s1074-7613(02)00365-5 PMC3689299

[19] Rescigno M (2011) Dendritic cells in bacteria handling in the gut. J Leukoc Biol 90: 669–672. 21771898 10.1189/jlb.0311141

[20] Farache J, Koren I, Milo I, Gurevich I, Kim KW, et al (2013) Luminal bacteria recruit CD103+ dendritic cells into the intestinal epithelium to sample bacterial antigens for presentation. Immunity 38: 581–595. 23395676 10.1016/j.immuni.2013.01.009 PMC4115273

[21] Niess JH, Adler G (2010) Enteric flora expands gut lamina propria CX3CR1+ dendritic cells supporting inflammatory immune responses under normal and inflammatory conditions. J Immunol 184: 2026–2037. 20089703 10.4049/jimmunol.0901936

[22] Ginhoux F, Liu K, Helft J, Bogunovic M, Greter M, et al (2009) The origin and development of nonlymphoid tissue CD103+ DCs. J Exp Med 206: 3115–3130. 20008528 10.1084/jem.20091756 PMC2806447

[23] Bogunovic M, Ginhoux F, Helft J, Shang L, Hashimoto D, et al (2009) Origin of the lamina propria dendritic cell network. Immunity 31: 513–525. 19733489 10.1016/j.immuni.2009.08.010 PMC2778256

[24] Vollstedt S, O'Keeffe M, Odermatt B, Beat R, Glanzmann B, et al (2004) Treatment of neonatal mice with Flt3 ligand leads to changes in dendritic cell subpopulations associated with enhanced IL-12 and IFN-alpha production. Eur J Immunol 34: 1849–1860. 15214033 10.1002/eji.200324443

[25] Ehigiator HN, Romagnoli P, Borgelt K, Fernandez M, McNair N, et al (2005) Mucosal cytokine and antigen-specific responses to Cryptosporidium parvum in IL-12p40 KO mice. Parasite Immunol 27: 17–28. 15813719 10.1111/j.1365-3024.2005.00736.x

[26] Ehigiator HN, McNair N, Mead JR (2007) Cryptosporidium parvum: the contribution of Th1-inducing pathways to the resolution of infection in mice. Exp Parasitol 115: 107–113. 16920103 10.1016/j.exppara.2006.07.001

[27] Iwasaki A, Kelsall BL (2000) Localization of distinct Peyer's patch dendritic cell subsets and their recruitment by chemokines macrophage inflammatory protein (MIP)-3alpha, MIP-3beta, and secondary lymphoid organ chemokine. J Exp Med 191: 1381–1394. 10770804 10.1084/jem.191.8.1381 PMC2193144

[28] Auray G, Lacroix-Lamande S, Mancassola R, Dimier-Poisson I, Laurent F (2007) Involvement of intestinal epithelial cells in dendritic cell recruitment during C. parvum infection. Microbes Infect 9: 574–582. 17395519 10.1016/j.micinf.2007.01.026

[29] Aiello S, Noris M, Piccinini G, Tomasoni S, Casiraghi F, et al (2000) Thymic dendritic cells express inducible nitric oxide synthase and generate nitric oxide in response to self- and alloantigens. J Immunol 164: 4649–4658. 10779769 10.4049/jimmunol.164.9.4649

[30] Leitch GJ, He Q (1999) Reactive nitrogen and oxygen species ameliorate experimental cryptosporidiosis in the neonatal BALB/c mouse model. Infect Immun 67: 5885–5891. 10531244 10.1128/iai.67.11.5885-5891.1999 PMC96970

[31] Fujimoto K, Karuppuchamy T, Takemura N, Shimohigoshi M, Machida T, et al (2011) A new subset of CD103+CD8alpha+ dendritic cells in the small intestine expresses TLR3, TLR7, and TLR9 and induces Th1 response and CTL activity. J Immunol 186: 6287–6295. 21525388 10.4049/jimmunol.1004036

[32] Barakat FM, McDonald V, Di Santo JP, Korbel DS (2009) Roles for NK cells and an NK cell-independent source of intestinal gamma interferon for innate immunity to Cryptosporidium parvum infection. Infect Immun 77: 5044–5049. 19687195 10.1128/IAI.00377-09 PMC2772539

[33] Dann SM, Wang HC, Gambarin KJ, Actor JK, Robinson P, et al (2005) Interleukin-15 activates human natural killer cells to clear the intestinal protozoan cryptosporidium. J Infect Dis 192: 1294–1302. 16136475 10.1086/444393

[34] Narni-Mancinelli E, Chaix J, Fenis A, Kerdiles YM, Yessaad N, et al (2011) Fate mapping analysis of lymphoid cells expressing the NKp46 cell surface receptor. Proc Natl Acad Sci U S A 108: 18324–18329. 22021440 10.1073/pnas.1112064108 PMC3215049

[35] Wyatt CR, Brackett EJ, Perryman LE, Davis WC (1996) Identification of gamma delta T lymphocyte subsets that populate calf ileal mucosa after birth. Vet Immunol Immunopathol 52: 91–103. 8807779 10.1016/0165-2427(95)05535-5 PMC7119672

[36] Korbel DS, Barakat FM, Di Santo JP, McDonald V (2011) CD4+ T cells are not essential for control of early acute Cryptosporidium parvum infection in neonatal mice. Infect Immun 79: 1647–1653. 21282414 10.1128/IAI.00922-10 PMC3067537

[37] Choudhry N, Petry F, van Rooijen N, McDonald V (2012) A protective role for interleukin 18 in interferon gamma-mediated innate immunity to Cryptosporidium parvum that is independent of natural killer cells. J Infect Dis 206: 117–124. 22517912 10.1093/infdis/jis300

[38] Zeng R, Oderup C, Yuan R, Lee M, Habtezion A, et al (2013) Retinoic acid regulates the development of a gut-homing precursor for intestinal dendritic cells. Mucosal Immunol 6: 847–856. 23235743 10.1038/mi.2012.123 PMC3612556

[39] Yeruva S, Ramadori G, Raddatz D (2008) NF-kappaB-dependent synergistic regulation of CXCL10 gene expression by IL-1beta and IFN-gamma in human intestinal epithelial cell lines. Int J Colorectal Dis 23: 305–317. 18046562 10.1007/s00384-007-0396-6 PMC2225996

[40] Chen XM, Levine SA, Splinter PL, Tietz PS, Ganong AL, et al (2001) Cryptosporidium parvum activates nuclear factor kappaB in biliary epithelia preventing epithelial cell apoptosis. Gastroenterology 120: 1774–1783. 11375958 10.1053/gast.2001.24850

[41] Zhou R, Gong AY, Eischeid AN, Chen XM (2012) miR-27b targets KSRP to coordinate TLR4-mediated epithelial defense against Cryptosporidium parvum infection. PLoS Pathog 8: e1002702. 22615562 10.1371/journal.ppat.1002702 PMC3355088

[42] Lacroix-Lamande S, Mancassola R, Auray G, Bernardet N, Laurent F (2008) CCR5 is involved in controlling the early stage of Cryptosporidium parvum infection in neonates but is dispensable for parasite elimination. Microbes Infect 10: 390–395. 18403229 10.1016/j.micinf.2007.12.020

[43] Wang HC, Dann SM, Okhuysen PC, Lewis DE, Chappell CL, et al (2007) High levels of CXCL10 are produced by intestinal epithelial cells in AIDS patients with active cryptosporidiosis but not after reconstitution of immunity. Infect Immun 75: 481–487. 17043107 10.1128/IAI.01237-06 PMC1828373

[44] Haniffa M, Shin A, Bigley V, McGovern N, Teo P, et al (2012) Human tissues contain CD141hi cross-presenting dendritic cells with functional homology to mouse CD103+ nonlymphoid dendritic cells. Immunity 37: 60–73. 22795876 10.1016/j.immuni.2012.04.012 PMC3476529

[45] Choudhry N, Korbel DS, Edwards LA, Bajaj-Elliott M, McDonald V (2009) Dysregulation of interferon-gamma-mediated signalling pathway in intestinal epithelial cells by Cryptosporidium parvum infection. Cell Microbiol 11: 1354–1364. 19473199 10.1111/j.1462-5822.2009.01336.x

[46] Vonarbourg C, Mortha A, Bui VL, Hernandez PP, Kiss EA, et al (2010) Regulated expression of nuclear receptor RORgammat confers distinct functional fates to NK cell receptor-expressing RORgammat(+) innate lymphocytes. Immunity 33: 736–751. 21093318 10.1016/j.immuni.2010.10.017 PMC3042726

[47] Middendorp S, Nieuwenhuis EE (2009) NKT cells in mucosal immunity. Mucosal Immunol 2: 393–402. 19587641 10.1038/mi.2009.99

[48] Moretto MM, Weiss LM, Combe CL, Khan IA (2007) IFN-gamma-producing dendritic cells are important for priming of gut intraepithelial lymphocyte response against intracellular parasitic infection. J Immunol 179: 2485–2492. 17675510 10.4049/jimmunol.179.4.2485 PMC3109618

[49] Chang JH, Cha HR, Chang SY, Ko HJ, Seo SU, et al (2011) IFN-gamma secreted by CD103+ dendritic cells leads to IgG generation in the mesenteric lymph node in the absence of vitamin A. J Immunol 186: 6999–7005. 21572021 10.4049/jimmunol.1003484

[50] Sun CM, Fiette L, Tanguy M, Leclerc C, Lo-Man R (2003) Ontogeny and innate properties of neonatal dendritic cells. Blood 102: 585–591. 12663436 10.1182/blood-2002-09-2966

[51] Scott CL, Aumeunier AM, Mowat AM (2011) Intestinal CD103+ dendritic cells: master regulators of tolerance? Trends Immunol 32: 412–419. 21816673 10.1016/j.it.2011.06.003

[52] Rescigno M (2010) Intestinal dendritic cells. Adv Immunol 107: 109–138. 21034972 10.1016/B978-0-12-381300-8.00004-6

[53] Lacroix-Lamande S, Mancassola R, Naciri M, Laurent F (2002) Role of gamma interferon in chemokine expression in the ileum of mice and in a murine intestinal epithelial cell line after Cryptosporidium parvum infection. Infect Immun 70: 2090–2099. 11895975 10.1128/IAI.70.4.2090-2099.2002 PMC127832

[54] Sawa S, Lochner M, Satoh-Takayama N, Dulauroy S, Berard M, et al (2011) RORgammat+ innate lymphoid cells regulate intestinal homeostasis by integrating negative signals from the symbiotic microbiota. Nat Immunol 12: 320–326. 21336274 10.1038/ni.2002

[55] Erridge C, Duncan SH, Bereswill S, Heimesaat MM (2010) The induction of colitis and ileitis in mice is associated with marked increases in intestinal concentrations of stimulants of TLRs 2, 4, and 5. PLoS One 5: e9125. 20161736 10.1371/journal.pone.0009125 PMC2817728

