# Supplementary figures and images for: Intestinal CD103+ Dendritic Cells Are Key Players in the Innate Immune Control of Cryptosporidium parvum Infection in Neonatal Mice
Source: PLoS Pathog. 2013 Dec 19;9(12):e1003801. doi: 10.1371/journal.ppat.1003801 (PMC3868524; doi:10.1371/journal.ppat.1003801)

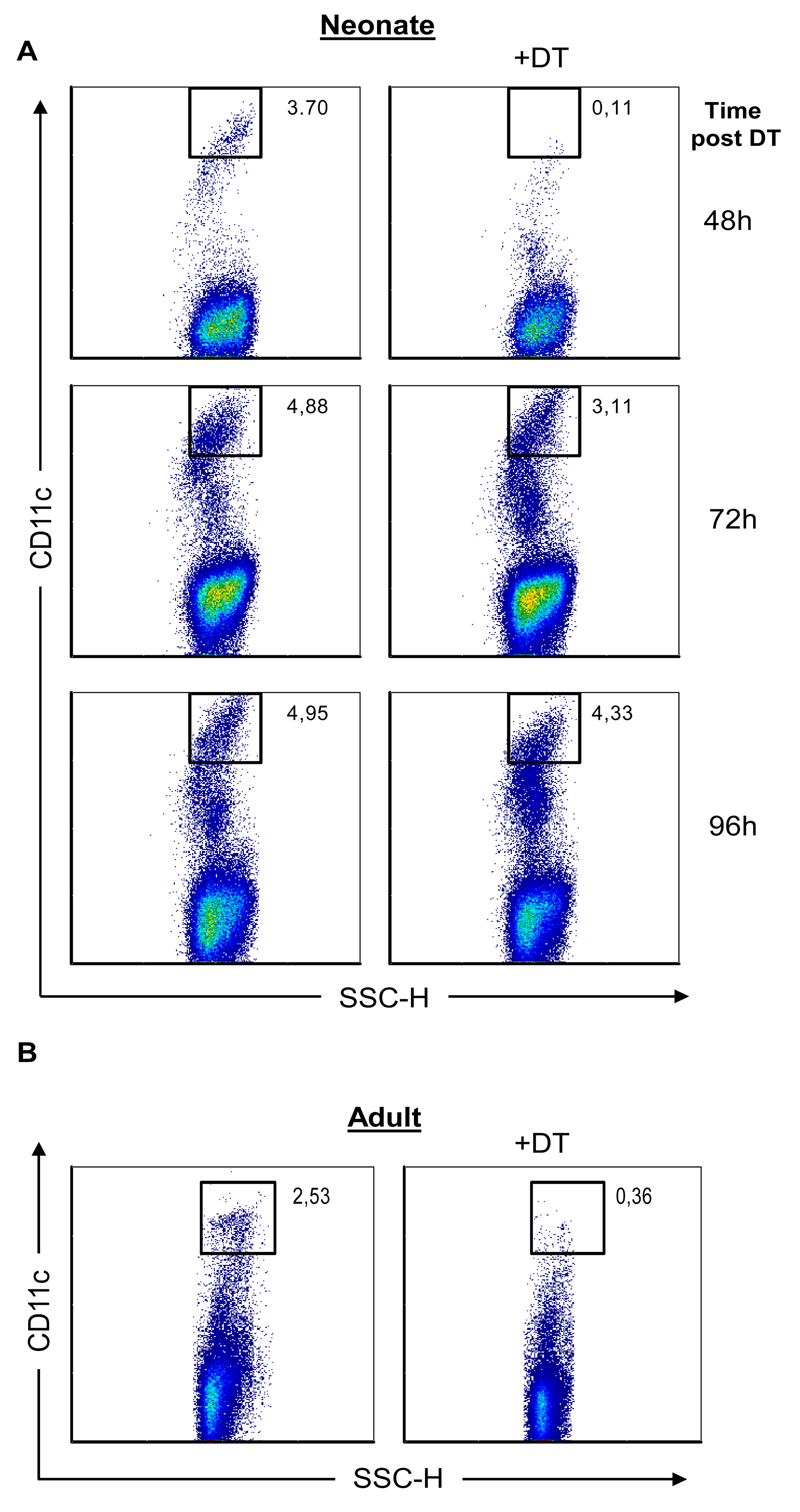

Supplement: Figure S1 — Restoration of CD11c+ cell counts in the infected mucosa of CD11c-DTR neonatal mice after transient depletion with DT. The presence of CD11c+ cells in the intestinal tissue was analyzed by flow cytometry. (A) Seven day-old heterozygous CD11c-DTR neonates were infected with 5.105 C. parvum oocysts and some animals were treated with DT 4 dpi. Intestinal cells were purified at 48 h, 72 h and 96 h post DT-treatment. (B) Adult CD11c-DTR animals were treated with DT and intestinal CD11c+ cells analyzed 24 h later. (TIF) [file ppat.1003801.s001.tif]

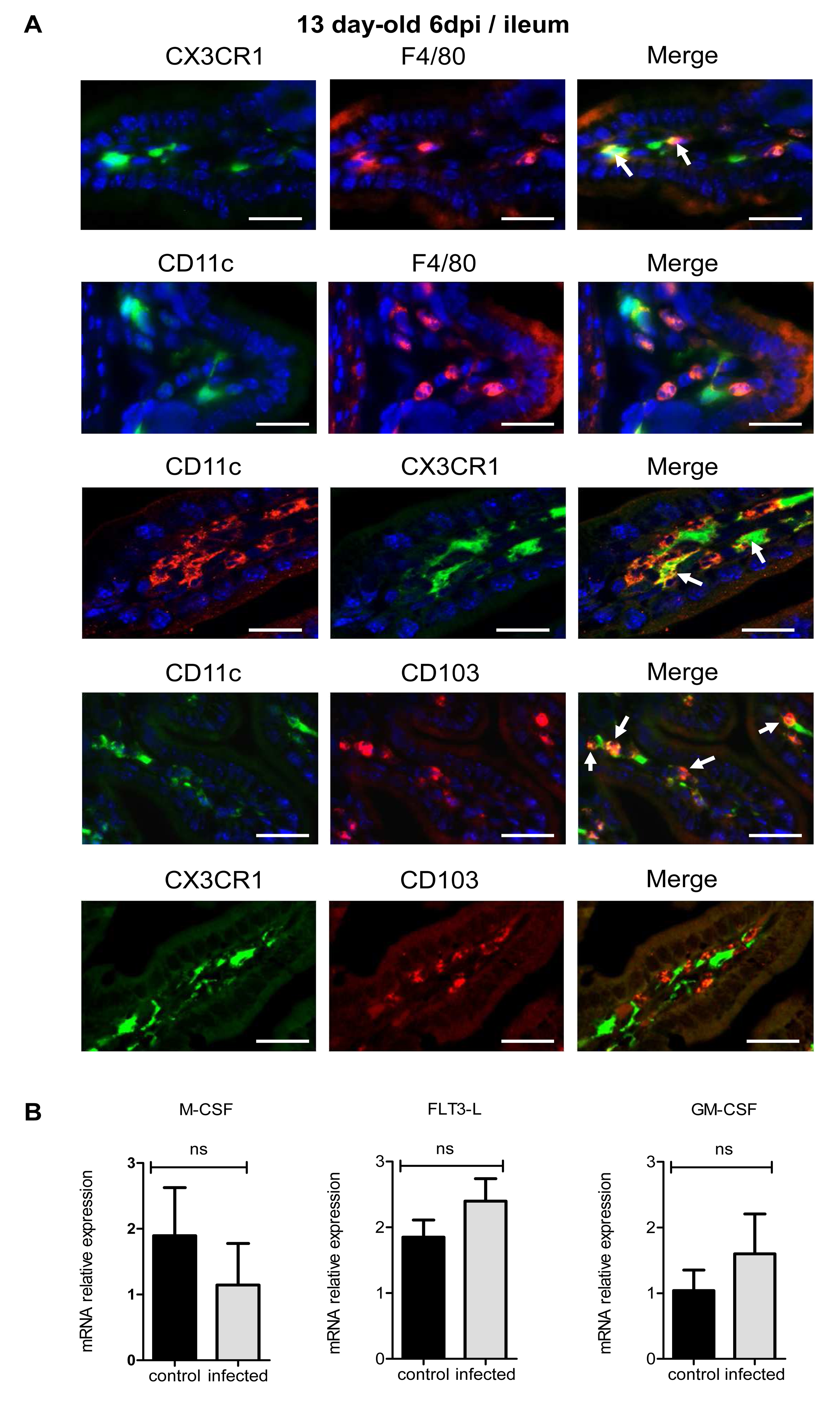

Supplement: Figure S2 — Mononuclear phagocyte populations in the intestinal mucosa of neonates at the peak of C. parvum infection. (A) Sections of the small intestine of infected neonates (6 dpi) were stained with Hoechst stain and antibodies against CD11c, CD103 and F4/80. CX3CR1GFP/+mice were used for CX3CR1 detection. The white arrows in the merge panel indicate double-positive cells. Intestinal CD103+ cells are distinct from F4/80+ cells such as CD11c+ cells and F4/80+ cells (Original magnification ×200; scale bars indicate 20 µm). (B) M-CSF, FLT3-L and GMCSF mRNAs were assayed in the ilea of 9 day-old neonates infected or not infected at 7 days of age. Data are means ± SEM of at least eight neonates in each group. Differences were not significant (ns) as assessed by Mann-Whitney non-parametric analyses (p values>0.05). (TIF) [file ppat.1003801.s002.tif]

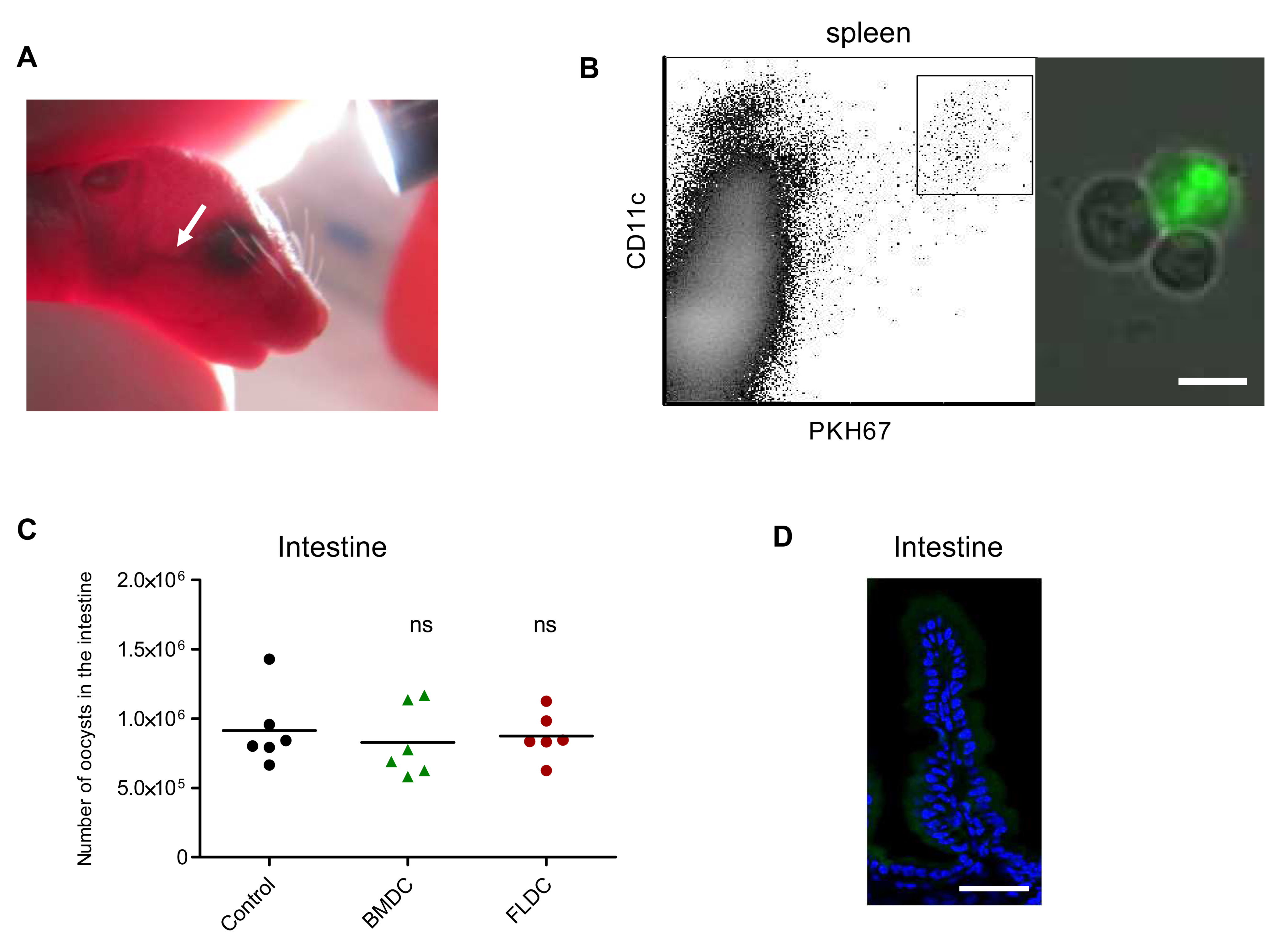

Supplement: Figure S3 — Dendritic cell transfer to neonates. (A) BMDC and FLDC were generated in vitro with GM-CSF and FLT3-L, respectively. Cells were injected by the intravenous route through the superficial temporal vein (see white arrow) according to Sands and Barker (Sands and Barker, 1999). Aliquots of 2×10^4^ stained BMDC were injected into day-old neonates; at that age, the skin is transparent, and the needle is visible through the skin. (B) For in vivo tracking of transferred cells, BMDC were stained with PKH67 and the presence of positive cells in the total spleen cell population of recipient neonates was analyzed 24 h (same results at 48 h) after the IV injection. The boxed region in the graph represents CD11c+ DC stained with PKH67 that have been transferred to recipient neonates. The image on the right-hand side shows a PKH67-stained cell adjacent to two unstained cells from a recipient neonate 24 h after transfer (scale bar indicates 10 µm). (C) Day-old littermate neonates were inoculated iv with 2×10^5^ BMDC or FLDC or mock inoculated. At seven days of age, the animals were all infected with 5×10^5^ oocysts of C. parvum and the parasite load in the intestine was evaluated 6 dpi. There was no significant difference between the groups. (D) To verify that BMDC efficiently migrated to the intestine, we tested for PKH67-BMDC in the intestine at the peak of infection by performing fluorescent microscopic analyses on sections. Despite extensive searching, no PKH67-BMDC were found in the infected intestine. Hoechst staining of the nucleus in blue (scale bar indicates 50 µm). Sands, M.S., and Barker, J.E. (1999). Percutaneous intravenous injection in neonatal mice. Lab Anim Sci 49, 328–330. (TIF) [file ppat.1003801.s003.tif]

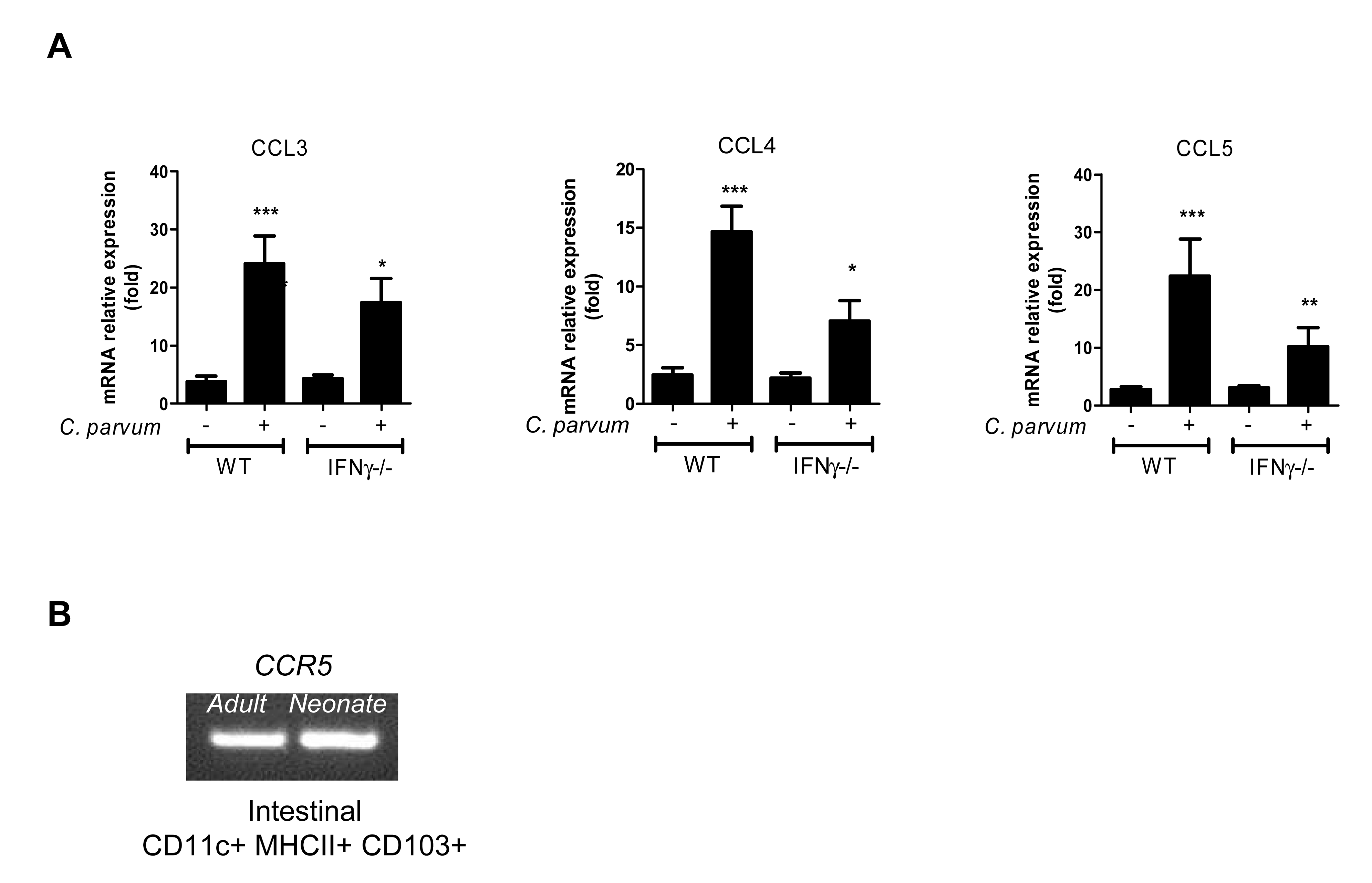

Supplement: Figure S4 — Expression of the chemokines CCL3, CCL4, CCL5 in IEC of infected IFNγ−/− neonatal mice, and expression of CCR5 by intestinal CD1O3+ DC. (A) Seven day-old C57BL/6J WT and IFNγ−/− neonates were infected with C. parvum. The mRNAs for CCL3, CCL4 and CCL5 in isolated IEC were assayed by qRT-PCR in infected (6 dpi) and in uninfected age-matched control neonates (n = 6 neonatal mice for each group,*** p<0.001, ** p<0.01, *p<0.05). (B) CD11c+ MHCII+ CD103+ DC isolated from the intestines of uninfected adults and infected neonates were sorted by flow cytometry. CCR5 expression in each sample was evaluated by RT-PCR. (TIF) [file ppat.1003801.s004.tif]

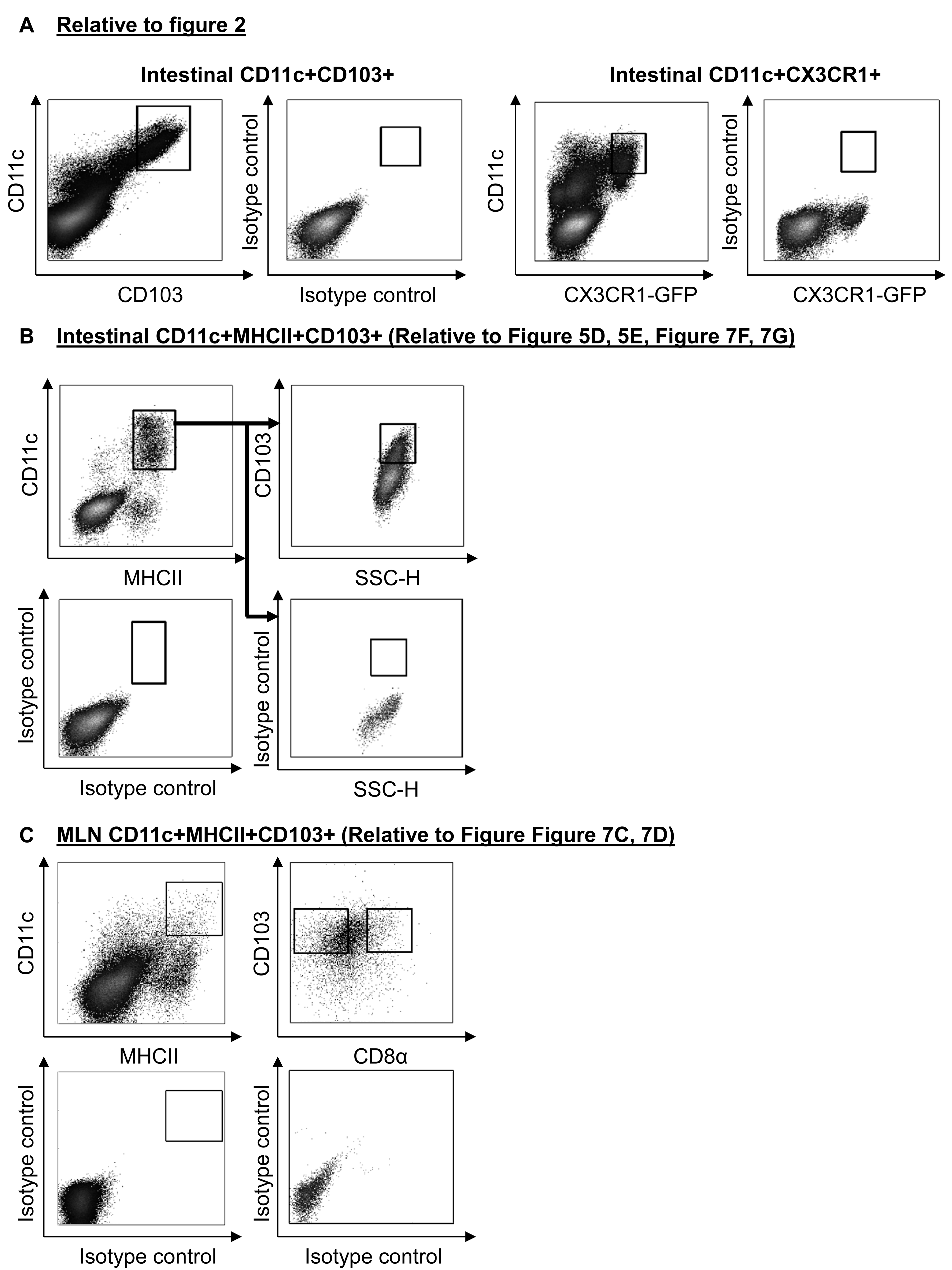

Supplement: Figure S5 — Gating strategies. (A) Gating strategies for cytometry analysis of intestinal CD11c+ CD103+ cells and CD11c+CX3CR1+ of Figure 2 (B) gating strategies used for flow cytometry analysis of intestinal CD103+ DC provided in figure 5D, 5E, 7F and 7G. CD103+ DC analysis was first based on selection of CD11chi MHC IIhi cells, which were then separated according to CD103 expression as indicated. (C) Gating strategies used for analysis of MLN CD103+DC subsets (relative to Figure 7C, 7D). Cells were first selected based on CD11chi MHC IIhi gating, then separated according to CD103 and CD8α expression as indicated. (TIF) [file ppat.1003801.s005.tif]
